# Olfactomedin 4 marks progenitor cells that give rise to the secretory cell lineage in the mouse cervix

**DOI:** 10.1242/dev.205710

**Published:** 2026-10-12

**Authors:** ShanmugaPriyaa Madhukaran, Yevgenia Fomina, Govindkumar Balagannavar, Elizabeth Payne, Jason Wilson, Lei Wang, Gary Hon, Maria Florian Rodriguez, Mala Mahendroo

**Affiliations:** ^1^ Department of Obstetrics and Gynecology, University of Texas Southwestern Medical Center, Dallas, TX 75390, USA; ^2^ Cecil H. and Ida Green Center for Reproductive Biology Sciences, University of Texas Southwestern Medical Center, Dallas, TX 75390, USA; ^3^ Department of Bioinformatics, University of Texas Southwestern Medical Center, Dallas, TX 75390, USA

**Keywords:** Cervix, Epithelia, Non-pregnant and pregnant cervix, Goblet cells, Olfactomedin 4, Female reproductive tract development, Mouse

## Abstract

Goblet cells, a specialized secretory cell type of mucosal epithelia, provide barrier function to the female lower reproductive tract. Current understanding proposes that squamous and secretory cells of the mouse cervicovaginal epithelium arise from a common Trp63^+^ progenitor. By contrast, in humans, a Krt8^+^ progenitor gives rise to endocervical secretory cells, whereas a Trp63^+^ progenitor gives rise to squamous cells in the ectocervix and vagina. We identify a population of Krt8^+^Trp63^−^ epithelia that is retained in the mouse cervicovaginal epithelium during postnatal development and adulthood, with olfactomedin 4 (Olfm4) as a specific marker of this lineage. Adult lineage tracing and marker assessment during postnatal development support a revised model in which two epithelial progenitors are delineated by postnatal day 15. Olfm4^+^ progenitors give rise to goblet cells, while Trp63^+^ progenitors give rise to keratinocytes in the cervicovaginal canal. Consistent with goblet cell expansion in pregnancy, Olfm4^+^ progenitors proliferate in early gestation and progesterone promotes their differentiation. These findings reveal a previously unrecognized similarity between mice and humans in which goblet and keratinized cell subtypes are derived from two independent progenitor populations.

## Teaser

**Highlighted Article:** Two distinct epithelial progenitors in the mouse cervix give rise to goblet cells or squamous cells, revealing an unexpected similarity to distinct progenitors in the human endocervix and ectocervix.

## INTRODUCTION

In the female reproductive tract (FRT), the cervical epithelium has a dual role; it facilitates sperm ascension necessary for fertilization and provides a protective mucosal barrier against ascending infections. During pregnancy, this barrier not only shields the maternal reproductive tract but also safeguards the fetus from infection and inflammation which can lead to preterm birth (PTB). In adults, cyclic hormonal changes drive the expansion of distinct cervical epithelial subtypes. One specialized subtype, the goblet cell, produces antimicrobials, protease inhibitors and immunoglobulin transporters along with gel-forming mucins such as MUC5B and MUC5AC. In both mice and humans, reduced expression of MUC5B has been associated with increased rates of PTB (Lacroix et al., 2022; Ueda et al., 2024). In mice, goblet cells expand during pregnancy and have been well defined by canonical regulators of secretory differentiation such as Spdef and Creb3l1 (Asada et al., 2012; Chen et al., 2009; Cooley et al., 2023; Noah et al., 2010). Although mucin-producing secretory cells are present in the human cervix (Gipson, 2001; Gipson et al., 1999), their identity as canonical goblet cells has not yet been established. Given the conserved importance of mucin-mediated epithelial defense across other mucosal organ systems (Kim and Ho, 2010; Nyström et al., 2021), understanding the developmental origin and regulation of cervical mucin-producing cells is essential for defining how the cervical epithelium contributes to reproductive barrier function and pregnancy maintenance.

The cervical epithelium is anatomically divided into the endocervix, which is continuous with the uterine epithelium, and the ectocervix, which transitions into the vaginal epithelium (see Table 1 for definitions of epithelial terminology). During early development in humans and mice, the cervical epithelium consists of a single-layered columnar epithelium marked by keratin 8 (KRT8). In humans, the endocervix remains columnar, with glandular development by 16-21 weeks of gestation, whereas the ectocervix differentiates into a multilayered, stratified squamous epithelium under the control of the transcription factor tumor protein 63 (TP63) (Cunha et al., 2017; Robboy et al., 2017). In mice, tissue recombination experiments and studies in Trp63 knockout (KO) mice demonstrate that signals from the underlying mesenchyme induce the Krt8^+^ columnar epithelium lining the neonatal cervix to express Trp63 between postnatal day (PND) 1 and PND5, driving epithelial stratification across both the endocervix and ectocervix (Fig. 1A) (Kurita, 2010; Kurita et al., 2005, 2004; Romano et al., 2012; Terakawa et al., 2020). Additionally, in humans, the vaginal and ectocervical epithelium arise from the urogenital ridge epithelium, while the endocervix and upper FRT arise from the Mullerian duct. In contrast, the entire mouse FRT, including the cervix and vagina, is derived from the Mullerian duct (Cunha et al., 2019).

**Table 1.** Definitions of epithelial terminology

Terms	Definition
Basal	A term that spatially defines the layers of cells close to the basement membrane. These cells express the basal marker Trp63.
Basal progenitor	These are a subset of Trp63^+^ basal cells that undergo proliferation and differentiate into luminal layers of stratified squamous epithelium.
Goblet cell	A specialized subtype of secretory epithelia that produces gel forming mucins (Muc5b, Muc5ac), transmembrane mucins (Muc1) and antimicrobial peptides necessary for mucosal barrier integrity. Distinct from other secretory cells, their differentiation is regulated by the transcription factor SPDEF.
Keratinocyte	Keratinocytes are the primary cell type of epidermis. The stratified squamous epithelial cells of the cervix are morphologically and transcriptionally similar to keratinocytes and thus are referred to as keratinocyte-like.
Luminal	A term that spatially defines the layer of epithelial cells that line the cervical lumen.
Olfm4 progenitor	These are subset of Olfm4^+^ cells with regenerative potential that give rise to goblet cells.
Secretory epithelium	Specialized epithelial tissue that synthesizes and releases secreted hormones or mucin proteins. Secretory epithelium can form glands or exist as a layer of cells. There are distinct subtypes of secretory cells in the female reproductive tract. Both luminal and glandular epithelium of the uterus are secretory cells that synthesize the transmembrane mucin protein, Muc1. The primary secretory cell in the cervix is the goblet cell.
Simple columnar or columnar	A one- or two-layer epithelium that is marked by expression of Krt8 and does not express Trp63.
Stratified squamous or squamous	Multi-layered epithelium derived from Trp63^+^ basal progenitors.

Developmental distinctions in epithelial origin may explain why the adult human endocervix remains columnar, whereas the adult mouse endocervix is stratified squamous. These species-specific differences raise the question of how distinct progenitor populations, stratified squamous progenitors in the mouse endocervix and columnar progenitors in the human endocervix, can give rise to similar secretory epithelial subtypes with conserved functions. While epithelial cell-fate plasticity in the FRT was previously thought to be restricted to the postnatal period, accumulating evidence indicates that bipotent stem cells persist in the uterus and cervix into adulthood and in the cervix can generate both squamous and columnar epithelial lineages (Jin, 2019; Zhao et al., 2021). Maintenance of epithelial cell fate in the adult uterus is regulated in part by a balance in estrogen and retinoic acid signaling (Parks et al., 2025; Rizo et al., 2023; Yin et al., 2025).

The focus of this study is to define the progenitor cell(s) that give rise to the cervical goblet cell. Leveraging our single-cell transcriptomic dataset from the mouse cervical epithelium, we delineate markers that distinguish goblet cells from other epithelial subtypes (Cooley et al., 2023). Among these, we identified olfactomedin 4 (Olfm4), a previously described marker of intestinal stem cells, as a distinguishing marker of cervical goblet cells (Schuijers et al., 2014). Using lineage tracing in reporter mice, we demonstrate the presence of a second progenitor population in the mouse cervix. This columnar progenitor is marked by Olfm4 at PND15 and gives rise to goblet cells in the adult mouse cervix and vagina. We propose the Olfm4^+^ columnar progenitor is distinct from Trp63^+^ basal progenitors, which give rise to stratified luminal subtypes. Our findings reveal a previously unreported progenitor lineage marker for goblet cells in the mouse cervix and vagina and highlight the epithelial diversity and plasticity of the adult lower FRT.

## RESULTS

### Identification of columnar and squamous markers in non-pregnant and pregnant mouse and human cervical epithelium

The adult mouse lower FRT epithelium consists primarily of a stratified squamous epithelium, in which a basal progenitor gives rise to cycle-dependent luminal cells (Fig. 1A) (Chateau and Boehm, 1996; Cunha et al., 2019; Forsberg, 1973; Kurita et al., 2005). Consistent with this model, our previous single-cell RNA-sequencing (scRNA-seq) studies identified luminal cell heterogeneity across non-pregnant (NP), pregnant and in-labor (IL) mice (Fig. 1B) (Cooley et al., 2023). The luminal populations are labeled as keratinocytes or clusters 1-6. Keratinocytes expressed the squamous cell marker Trp63 and keratinocyte cell markers Dsg1a^+^, Dmkn^+^, Krt16^+^ and Krt12^+^. Clusters 1-6 were designated as secretory based on expression of either the transmembrane mucin Muc1, or gel-forming mucin Muc5b, as shown in the dot plot in Fig. 1C. Secretory clusters 1-6 uniquely expressed the columnar cell marker Krt8. Secretory clusters 2-6 also did not express the squamous cell marker Trp63. Olfm4 was uniquely enriched in secretory clusters 2, 3, 5 and 6 with little to no expression in clusters 1 and 4. Clusters 1,2, 4, 5 and 6 expressed the goblet cell markers SAM pointed domain containing ETS factor (SPDEF), a transcription factor required for goblet cell differentiation (Cooley et al., 2023; Noah et al., 2010) and Muc5b. Cluster 3, predominant in NP cervix, only expressed the transmembrane mucin Muc1. We have recently demonstrated that goblet cells are the primary luminal cells lining the mouse endocervix and ectocervix during pregnancy, and in labor, and they are present in lower abundance during the diestrus phase of the reproductive cycle (Cooley et al., 2023). Previous studies in pregnant individuals and mice suggest that goblet cell dysfunction disrupts the mucosal barrier and increases risk of ascending infection-mediated PTB (Lacroix et al., 2022; Ueda et al., 2022). To better understand the cell states of the secretory clusters and lineage trajectories that give rise to goblet cells, we characterized this functionally important subtype using markers that distinguish them from other epithelial subtypes.

**Fig. 1.**
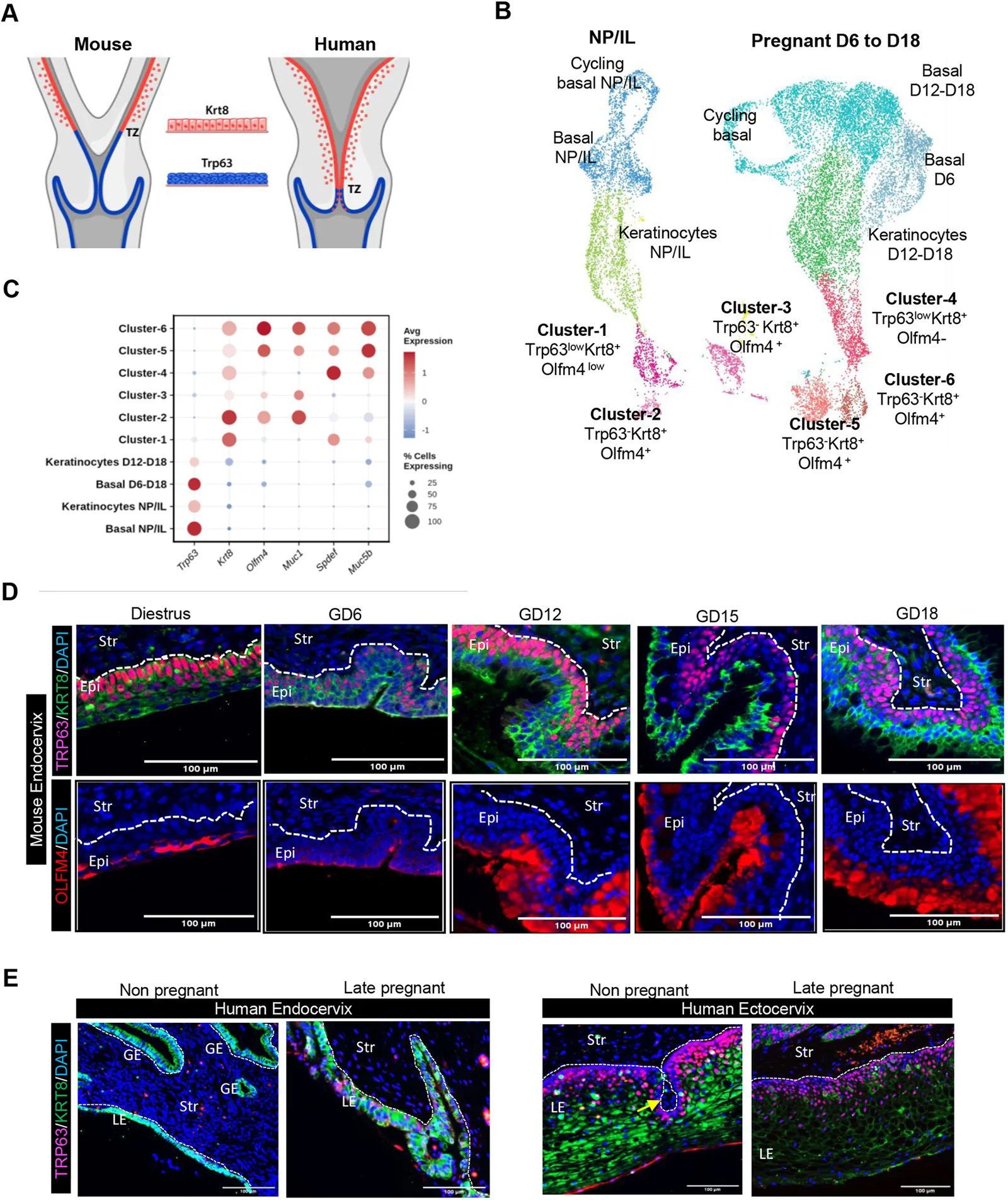
**Expression of squamous and columnar epithelial populations in the mouse and human cervix across pregnant and non-pregnant states.** (A) Schematic comparison of the human and mouse female reproductive tract (FRT), highlighting the luminal (red line) and glandular (red circles) columnar epithelial organization of the uterus, and the distinct columnar and squamous (blue line) epithelial regions between the mouse and human cervix, with overlap of these subtypes localized at the transition zone (TZ). (B) UMAP visualization of mouse cervical epithelium across reproductive states at non-pregnant (estrus and diestrus; NP), pregnant (GD6, GD12, GD15, GD18) and in-labor (IL) timepoints. Epithelial subtypes are color-coded to indicate basal, keratinocyte or secretory clusters 1-6 (modified from Cooley et al., 2023). (C) Dot plots indicate the percentage of cells in each cluster expressing the genes *Trp63*, *Krt8*, *Olfm4*, *Muc1*, *Spdef* and *Muc5b*. Color indicates scaled average expression. (D) Immunofluorescence detection of TRP63 (red), KRT8 (green), nuclei (blue) and OLFM4 (red) in mouse endocervical epithelium at NP (diestrus), pregnant (GD6, GD12, GD15, GD18) timepoints. Corresponding images of ectocervix and vaginal regions from the same animals are shown in Fig. S1A. *n*≥3 biological replicates per timepoint. (E) Immunofluorescence detection of TRP63 (red), KRT8 (green) and nuclei (blue) in human non-pregnant and late-pregnant (34 weeks gestation) cervix. Images of endocervix and ectocervix regions are from the same individual; yellow arrow indicates stromal papillae. Dashed lines show the boundary between epithelia and stroma. Epi, epithelial region; GE, glandular epithelia; LE, luminal epithelia; Str, stromal region. *n*≥3 biological replicates per group. Scale bars: 100 μm.

The spatial distribution of TRP63, KRT8 and OLFM4 proteins was assessed in the mouse endocervix, ectocervix and vagina across NP diestrus and pregnancy gestational days (GD) 6, 12, 15 and 18 (Fig. 1D; Fig. S1A). TRP63^+^ cells were confined to the endocervical, ectocervical and vaginal basal layer adjacent to the stroma across all stages. KRT8^+^ cells were localized to luminal cell layers during diestrus and in pregnancy (Fig. 1D; Fig. S1A). On GD6, a few TRP63^+^ KRT8^+^ double positive cells were present in the basal layer, yet these steadily declined in the later pregnancy timepoints. OLFM4 expression mirrored KRT8 expression in the luminal epithelial layers across all stages, consistent with the spatial localization and temporal pattern of goblet cells (Fig. 1D; Fig. S1A).

We next performed a parallel analysis in the human endocervix and ectocervix from premenopausal or third trimester pregnant individuals (Fig. 1E). Both luminal and glandular epithelia of the endocervix were KRT8^+^ consistent with columnar cells. Interestingly, a few endocervical cells were also TRP63^+^ in the pregnant cervix. The basal layers of the ectocervix were TRP63^+^ in both the NP and pregnant samples. The luminal layers were KRT8^+^ with some TRP63 KRT8 co-positive cells. In contrast to mice, OLFM4 protein expression was not detectable in the human endocervix or ectocervix (Fig. S1B).

### OLFM4^+^ luminal cells undergo proliferation in early pregnancy

Olfm4 marks epithelial progenitor/stem cells in the intestine (Sakahara et al., 2024; Schuijers et al., 2014; van der Flier et al., 2009) and prostate (Li et al., 2020) where Olfm4^+^ cells contribute to tissue regeneration and maintenance. In the mouse cervix, immunofluorescent co-staining of OLFM4 with the proliferation marker KI67 and epithelial marker E-cadherin, CDH1, identified proliferative OLFM4^+^ cells in luminal layers during secretory cell expansion on GD6 and to a lesser extent in diestrus (Fig. S1C). In contrast, in estrus and late pregnancy (GD15), proliferating cells were confined to the basal layer of both the endocervix and ectocervix, suggesting that basal cells are the predominant proliferative population at these stages. In early pregnancy (GD6), proliferation extended across all epithelial layers in both regions (Fig. S1C). The presence of stage-specific proliferative OLFM4^+^ cells in the cervix, together with the established regenerative potential of OLFM4-marked cells in other tissues, led us to hypothesize that OLFM4 marks a luminal progenitor population in the cervical epithelium.

### Olfm4 expression is initiated during cervical postnatal development in mice

We sought to determine whether Olfm4 is expressed during postnatal development of the FRT. To define the temporal pattern of OLFM4 expression during cervical development, we assessed its expression in combination with established markers, KRT8 (columnar), TRP63 (squamous) and *Muc1* (secretory) at key postnatal timepoints in FRT development: PND1, PND5, PND15 and PND25 (Fig. 2A and Fig. S2).

**Fig. 2.**
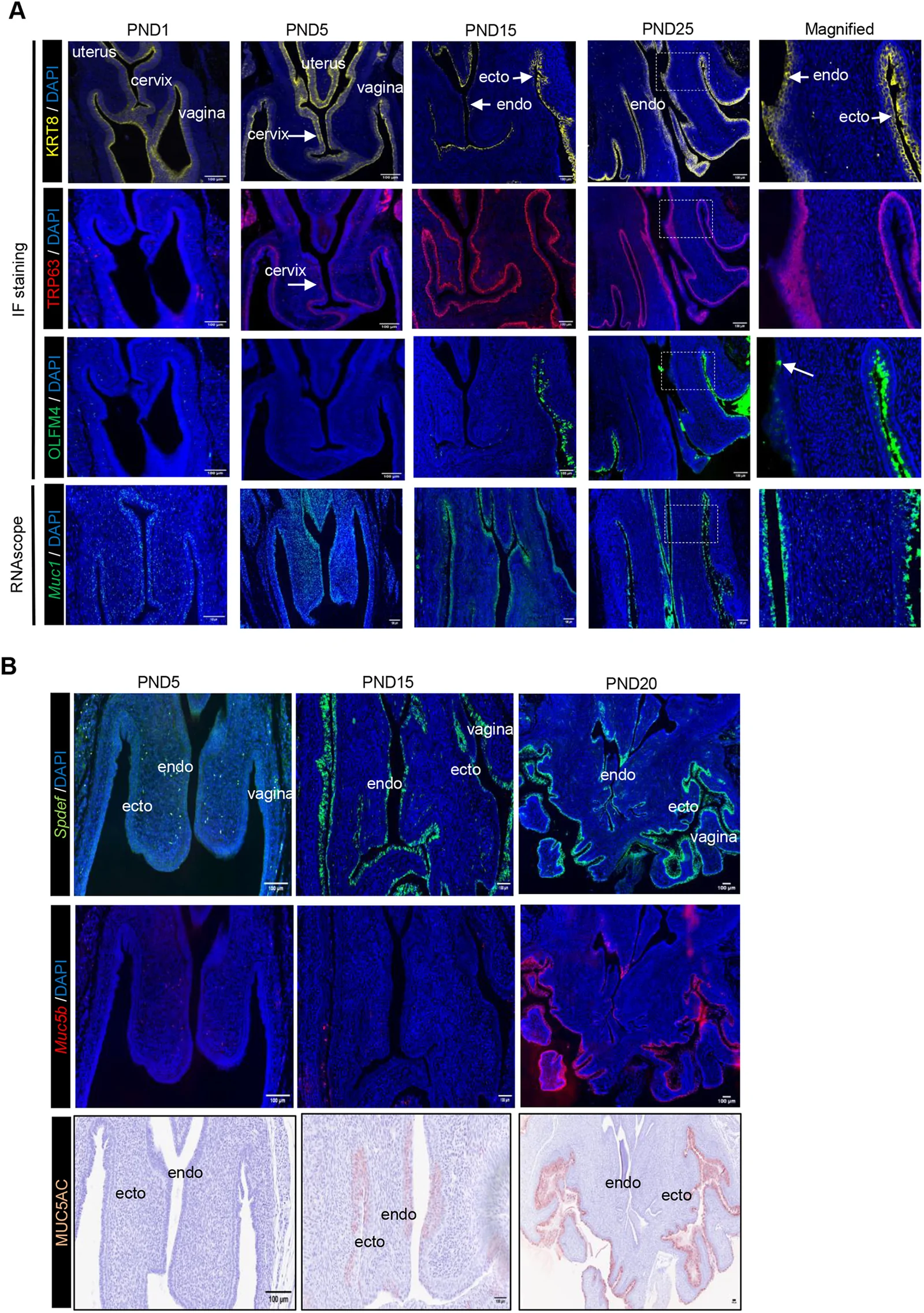
**Olfm4 marks luminal cervical epithelia during postnatal FRT development.** (A) Immunofluorescence staining of the female reproductive tract (FRT) for KRT8 (yellow), TRP63 (red), OLFM4 (green) and RNA expression of *Muc1* (green) at PND1, PND5, PND15 and PND25. The right-most panels shows a higher-magnification view of the PND25 cervix, highlighting spatial restriction of OLFM4 to the luminal columnar cells in the endocervix (endo), ectocervix (ecto) and vagina (white arrows). Except for *Muc1*, serial sections from the same animal were used for each panel at each timepoint, with *n*=3 biological replicates per timepoint. (B) RNA expression of *Spdef* and *Muc5b* performed on the same tissue section (signals displayed in separate panels for visualization). Immunostaining for MUC5AC protein in the FRT at PND5, PND15 and PND20 (*n*=2 biological replicates per timepoint). Scale bars: 100 μm.

On PND1, the cervical epithelial layers were KRT8^+^ epithelia and expressed *Muc1* transcripts. By PND5, all the multilayered cervical epithelium was KRT8^+^ and the basal layer of cells co-expressed TRP63. *Muc1*, in the uterus and cervix overlapped with KRT8. Since OLFM4 expression was absent at both PND1 and PND5, it does not appear to be an early marker of cervical epithelial differentiation. Between PND15 and PND25, TRP63 expression was predominant in the basal layers of epithelial cells, while a thin luminal layer of cells remained KRT8^+^*Muc1*^+^. Among the KRT8^+^ luminal cells, a subset expressed OLFM4 in the endocervix, with a greater number of KRT8^+^OLFM4^+^ cells in the ectocervix and vaginal epithelia (Fig. 2A; Fig. S2B). The KRT8^+^OLFM4^+^ cells also expressed goblet cell markers including *Spdef* and the goblet cell-specific mucins, *Muc5b* and MUC5AC (Fig. 2B), suggesting that goblet progenitor specification is established by PND15. These findings support a stepwise delineation of two distinct epithelial progenitor populations in the postnatal period with basal-localized TRP63^+^ and a luminal-localized KRT8^+^OLFM4^+^ population. In contrast to the cervix, the expression of Olfm4 and goblet cell markers are not detected in the uterine epithelium in the postnatal period (Fig. S2C). This observation is consistent with published scRNA-seq datasets of the uterus between PND1 and PND15, in which <2.6% of cells had detectable cell counts for Olfm4 or Spdef (Fig. S2D; Spencer et al., 2023).

### Krt8^+^ progenitors give rise to goblet cells in the cervix and vagina

Given the observation that the luminal layers of cervical epithelia do not gain Trp63 expression during the FRT developmental period and do express Krt8, we tested the potential of a Krt8^+^ progenitor cell to give rise to goblet cells in the adult mouse cervix before and during pregnancy. Lineage-tracing studies were conducted in the Krt8-CreERT2; Rosa26tdTomato females as depicted in Fig. 3A. In the cervix and vagina, tdTomato^+^ cells were primarily confined to luminal layers. The tdTomato^+^ cells expressed the goblet cell markers Spdef and Muc5b during diestrus and pregnancy timepoints, confirming that Krt8^+^ progenitor cells give rise to goblet cells (Fig. 3B; Fig. S3). These findings support the presence of a cervical columnar Krt8^+^ progenitor as the precursor for goblet cells.

**Fig. 3.**
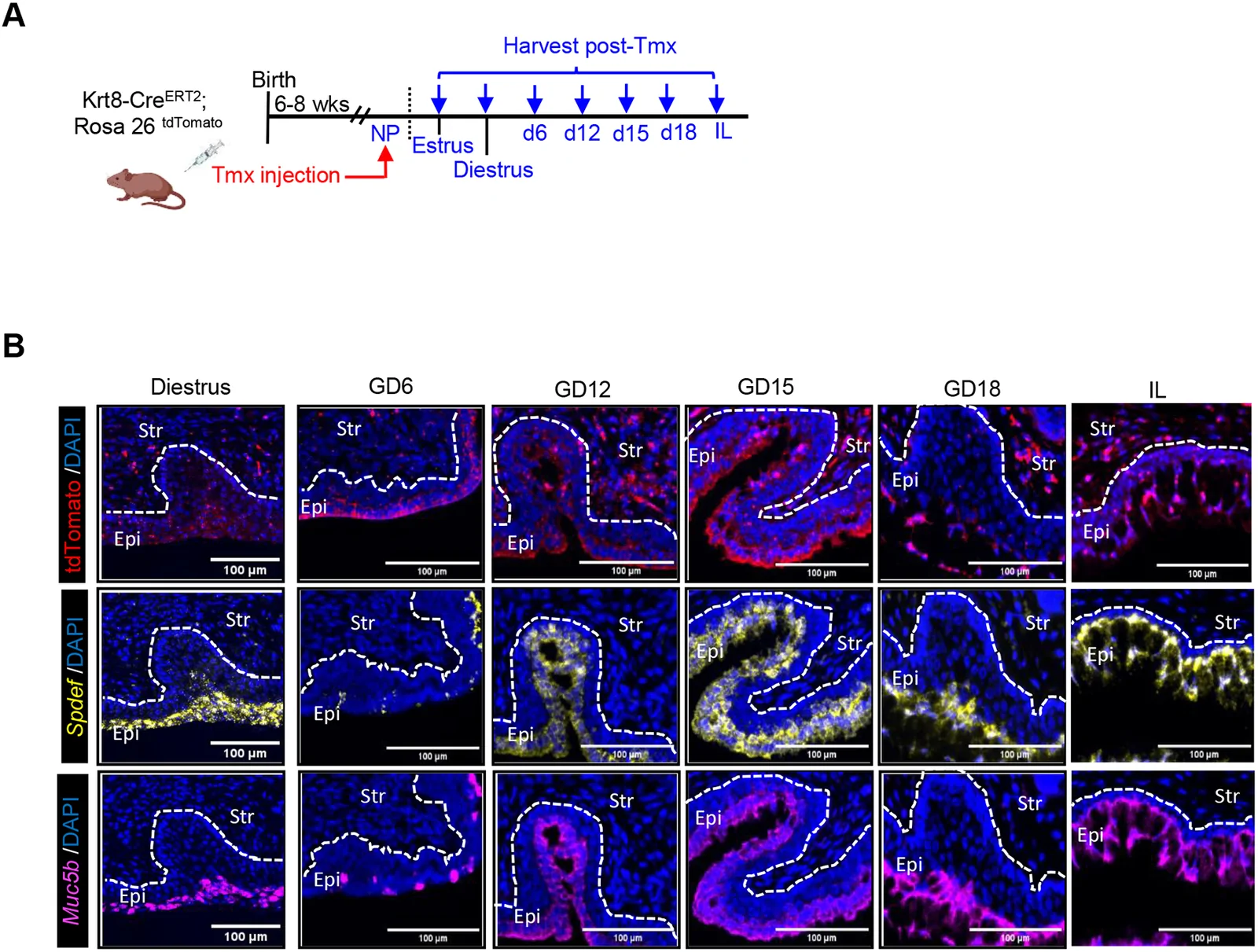
**Krt8^+^ progenitor cells give rise to goblet cells in the cervix.** (A) Schematic of lineage-tracing strategy using Krt8-Cre^ERT2^; Rosa26^tdTomato^ mice with tissues collected across non-pregnant and early- to late-pregnancy [gestation days (d) 6, 12, 15 and 18] and in labor (IL). (B) RNA expression in the endocervix for tdTomato (red), *Spdef* (yellow) and *Muc5b* (pink) across diestrus, pregnancy stages and in labor. *Spdef* and *Muc5b* were co-stained in the same section, with each marker displayed in separate panels, whereas tdTomato was detected on a separate section. Epithelial (Epi) and stromal (Str) regions are indicated. Dashed lines show the boundary between epithelia and stroma. Images are representative of *n*=1 biological replicate per timepoint. Scale bars: 100 μm.

### Olfm4^+^ luminal progenitors represent a self-renewing epithelial population that give rise to cervical goblet cells

To evaluate the progenitor potential of Olfm4-expressing cells in the cervix, we conducted lineage-tracing studies using Olfm4-IRES-eGFPCre^ERT2^;Rosa26^tdTomato^ reporter mice (Schuijers et al., 2014). NP Olfm4Cre^ERT2^; R26^tdTomato^ mice received a single intraperitoneal injection of tamoxifen (Tmx; 1 mg in 100 µl corn oil) or corn oil (Fig. 4A). Following Tmx induction, mice were either maintained as NP or timed-mated, and the cervical tissues were collected for spatial assessment of tdTomato expression and lineage-specific mRNA or protein markers. Reporter specificity was established by Olfm4 and tdTomato co-staining (Fig. S4A) and the quantification of flow-sorted tdTomato^+^ cells in corn oil versus Tmx-treated Olfm4Cre^ERT2^; R26^tdTomato^ mice. In NP diestrus mice treated with Tmx, 33% of epithelia (EpCAM^+^) were tdTomato^+^ in comparison to 0.04% in the control group (Fig. S4B). Similarly, in GD15 pregnant mice, 26% of epithelia were tdTomato^+^ compared to 0.01% in the control group. At all timepoints during and outside of pregnancy and non-pregnancy, tdTomato^+^ cells were restricted to the luminal layer of the endocervix, ectocervix and vagina (Fig. 4B; Fig. S4C,D), indicating that these cells do not arise from a basal progenitor population but instead originate from an Olfm4^+^ progenitor compartment.

**Fig. 4.**
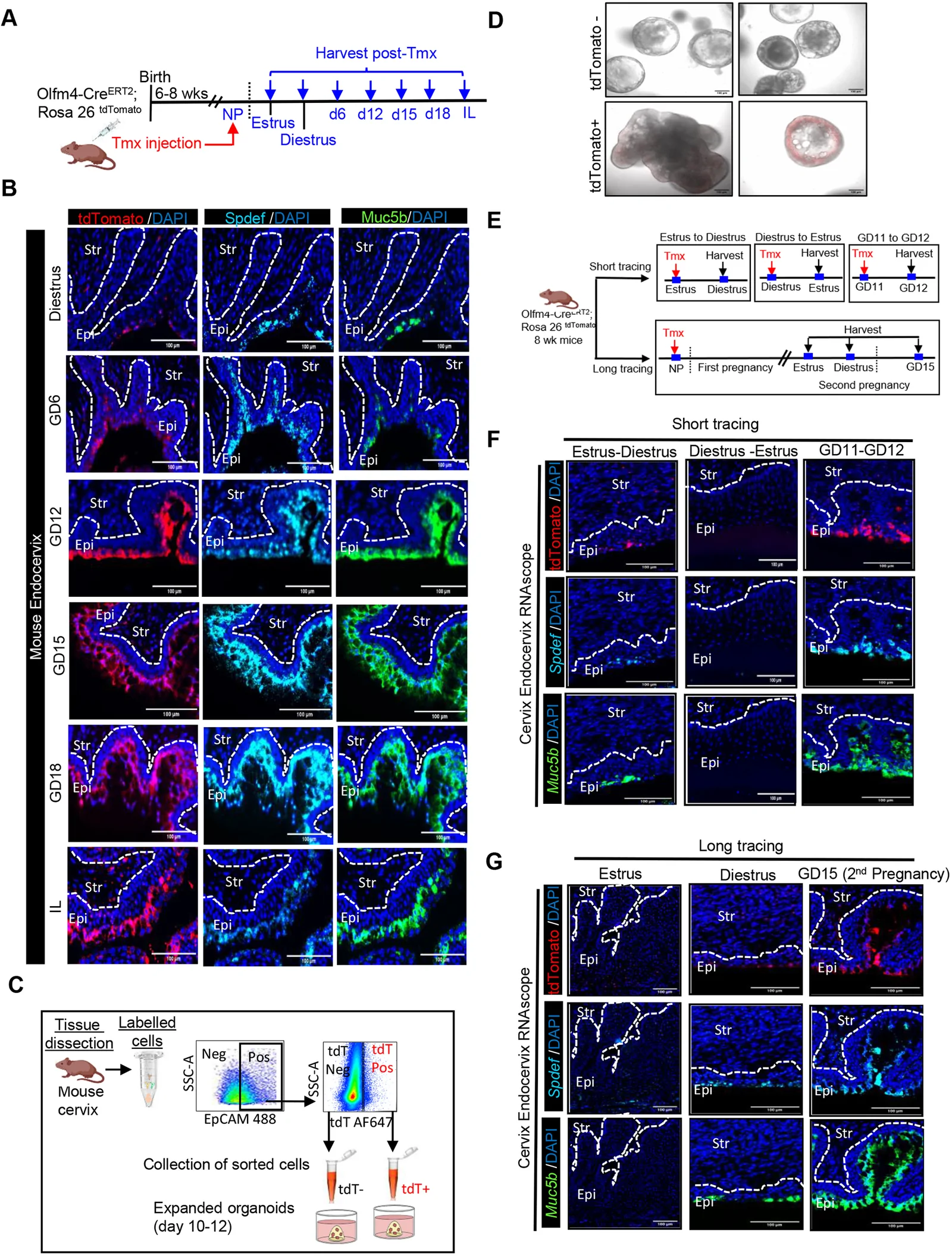
**OLFM4^+^ progenitors represent a self-renewing epithelial population that give rise to cervical goblet cells.** (A) Schematic of lineage-tracing strategy using Olfm4-Cre^ERT2^; Rosa26^tdTomato^ mice. (B) RNA expression of tdTomato^+^ (red), Spdef (cyan) and Muc5b (green) in endocervical tissue from non-pregnant mice (diestrus; NP) and during pregnancy (GD6, GD12, GD15, GD18 and IL). All three markers were detected in the same section, with each signal displayed in separate panels. Images are representative of *n*≥3 biological replicates per timepoint. (C) Schematic of workflow used to generate organoid cultures from tdTomato^−^ and tdTomato^+^ epithelial populations from the cervix of NP diestrus Olfm4-Cre^ERT2^; Rosa26^tdTomato^ mice. (D) Organoids generated from flow cytometry sorted tdTomato^+^ and tdTomato^−^ epithelial populations. tdTomato^+^-derived organoids retained tdTomato signal, whereas tdTomato^−^ progenitors formed organoids that did not express tdTomato. *n*=3 biological replicates. (E) Schematic of short-term and long-term lineage-tracing strategy using Olfm4 Cre^ERT2^; Rosa26^tdTomato^ mice (8-week-old females). Short-term tracing (top): Tamoxifen (Tmx) was administered at estrus, diestrus or GD11, and tissues were collected 24-48 h later at the subsequent timepoints (estrus to diestrus, diestrus to estrus, GD11 to GD12). Long-term tracing (bottom): Tmx was administered at the NP stage, followed by a first pregnancy. After the first pregnancy, tissues were collected at two stages of the estrous cycle (estrus and diestrus) or on GD15 of a second pregnancy. (F) RNAscope images of endocervix from short-term tracing experiments. Panels indicate tdTomato^+^ (red), Spdef (cyan), Muc5b (green) and nuclei (blue). In short-term tracing, tdTomato^+^ cells are present and co-localize with goblet markers during diestrus (first column) and on GD12 (third column). All three markers were stained in the same section and are displayed in separate panels. Representative images from *n*=2 biological replicates per condition. (G) RNAscope images of endocervix from long-term tracing experiments. Panels indicate tdTomato^+^ (red), Spdef (cyan), Muc5b (green) and nuclei (blue). In long-term tracing, tdTomato^+^ cells persist and give rise to goblet cells that express Spdef and Muc5b in diestrus (second column) and in a second pregnancy at GD15 (third column). All three markers were stained in the same section and are displayed in separate panels. Representative images from *n*=2 biological replicates per condition. Epithelial (Epi) and stromal (Str) regions are indicated. Dashed lines show the boundary between epithelia and stroma. Scale bars: 100 μm.

A gradual increase in luminally localized tdTomato^+^ cells was observed from GD6 to GD18 and in labor in Tmx-treated mice (Fig. 4B). RNAscope analysis also demonstrated overlap of cervical tdTomato with *Spdef* as well as the secretory mucin *Muc5b*, providing further support that Olfm4^+^ progenitor cells give rise to the goblet cells (Fig. 4B). As an orthogonal approach, organoid cultures were derived from flow-sorted tdTomato^+^ and tdTomato^−^ cervical epithelial cells (Fig. 4C). Organoids derived from tdTomato^+^ cells consistently retained tdTomato fluorescence and expression over multiple passages, whereas organoids derived from tdTomato^−^ cells remained negative (Fig. 4D). Despite the demonstration that organoids are derived from progenitor cells with lineage specificity, organoids from both tdTomato^+^ or tdTomato^−^ progenitors expressed TRP63^+^ cells and KRT8^+^ cells (Fig. S4E). This suggests cellular plasticity under the organoid culture conditions and warrants future investigation. Organoid growth efficiency was reduced in the tdTomato^+^ as compared to tdTomato^−^ organoids (Fig. S4F). Together, the lineage-tracing data identify an Olfm4^+^ luminal progenitor population in the cervix that undergoes marked expansion early in pregnancy and subsequently differentiates into goblet cells throughout gestation.

Our previously reported single-cell transcriptomic dataset from NP mice was generated from pooled estrus and diestrus samples (Cooley et al., 2023). Within this dataset, two secretory epithelial populations were identified. One population was classified as canonical goblet cells, while the second was designated a non-goblet secretory population based on low Spdef expression and absence of Muc5b expression (Fig. 1B) (Cooley et al., 2023). To distinguish between two secretory cell types or transition states, Olfm4Cre^ERT2^; R26^tdTomato^ mice were assessed at proliferative (estrus), early secretory (metestrus) and secretory (diestrus) stages of the NP cycle for the presence of tdTomato^+^ cells and for *Spdef*, *Muc5b* and *Muc1* expression in the endocervix (Fig. S4G). Consistent with their progenitor potential, tdTomato^+^ cells were present at all three stages of the cycle, with the highest abundance observed in diestrus. Integrated analysis of secretory marker expression across phases suggests a stepwise differentiation of Olfm4^+^ progenitors from an early/intermediate in estrus/metestrus to fully differentiated goblet cells in diestrus. Thus, the cluster identified as ‘secretory’ in Fig. 1B is likely an early goblet cell cluster. This dynamic differentiation program positions Olfm4^+^ progenitors as a shared unipotent progenitor pool for cervical goblet cells during both non-pregnancy and pregnancy.

In addition to the above-described regime for activation of the Cre reporter, tdTomato^+^ cells that express goblet cell markers were observed after short term (24-28 h) and with long term (second pregnancy) tracing in which tdTomato^+^ cells continued to give rise to goblet cells in a second pregnancy (Fig. 4E-G). Reporter specificity after short tracing is supported by co-staining of tdTomato and Olfm4 (Fig. S4H). Collectively, these findings support the conclusion that Olfm4^+^ luminal progenitors represent a self-renewing epithelial population with a sustained capacity to generate cervical goblet cells across the reproductive cycle.

### Olfm4 is not required for goblet cell development

In addition to marking Lgr5^+^ intestinal stem cells, Olfm4 is a glycoprotein produced by epithelia and neutrophils with conserved roles in mucosal immunity across tissues, including the gastrointestinal and respiratory tracts (Al Gharaibeh et al., 2023; Alder et al., 2019; Clemmensen et al., 2012; Gersemann et al., 2012; Liu and Rodgers, 2016; Liu et al., 2013). As Olfm4 expression is initiated postnatally and upregulated in the mouse cervix during pregnancy (Fig. 1D), we asked whether Olfm4 is also required for cervical goblet cell differentiation and mucosal barrier function. Using mice with a targeted Olfm4 deletion (Liu et al., 2013), histologic analyses of NP and pregnant mice (GD6-GD18) revealed morphologically similar secretory cells in the endocervix (Fig. 5A; Fig. S5A). Consistent with this, expression of goblet cell transcription factor Spdef and mucin Muc5b were comparable between genotypes (Fig. 5B; Fig. S5B), indicating that Olfm4 is not required for cervical goblet cell differentiation. To assess barrier function in Olfm4-deficient mice, we introduced an ascending vaginal infection with pathogenic *Escherichia coli* (serotype O55; ATCC) at GD15. Preterm birth rates were not significantly different between wild-type (WT) and Olfm4-deficient mice (*P*=0.198, Fisher's exact test) (Table S1). Next, we used bioluminescent *E. coli* to track bacterial ascension (Alder et al., 2019), with WT and Olfm4-deficient mice showing similar vaginal colonization at 0 h and equivalent spread of *E. coli* to the uterus at 12 h post-inoculation (Fig. 5C). Inflammatory responses were further evaluated by intraperitoneal lipopolysaccharide (LPS) administration (5 mg/kg) at GD15. No significant differences were observed in the expression of proinflammatory genes in the cervix (top panel) or uterus (bottom panel) (Fig. S5C).

**Fig. 5.**
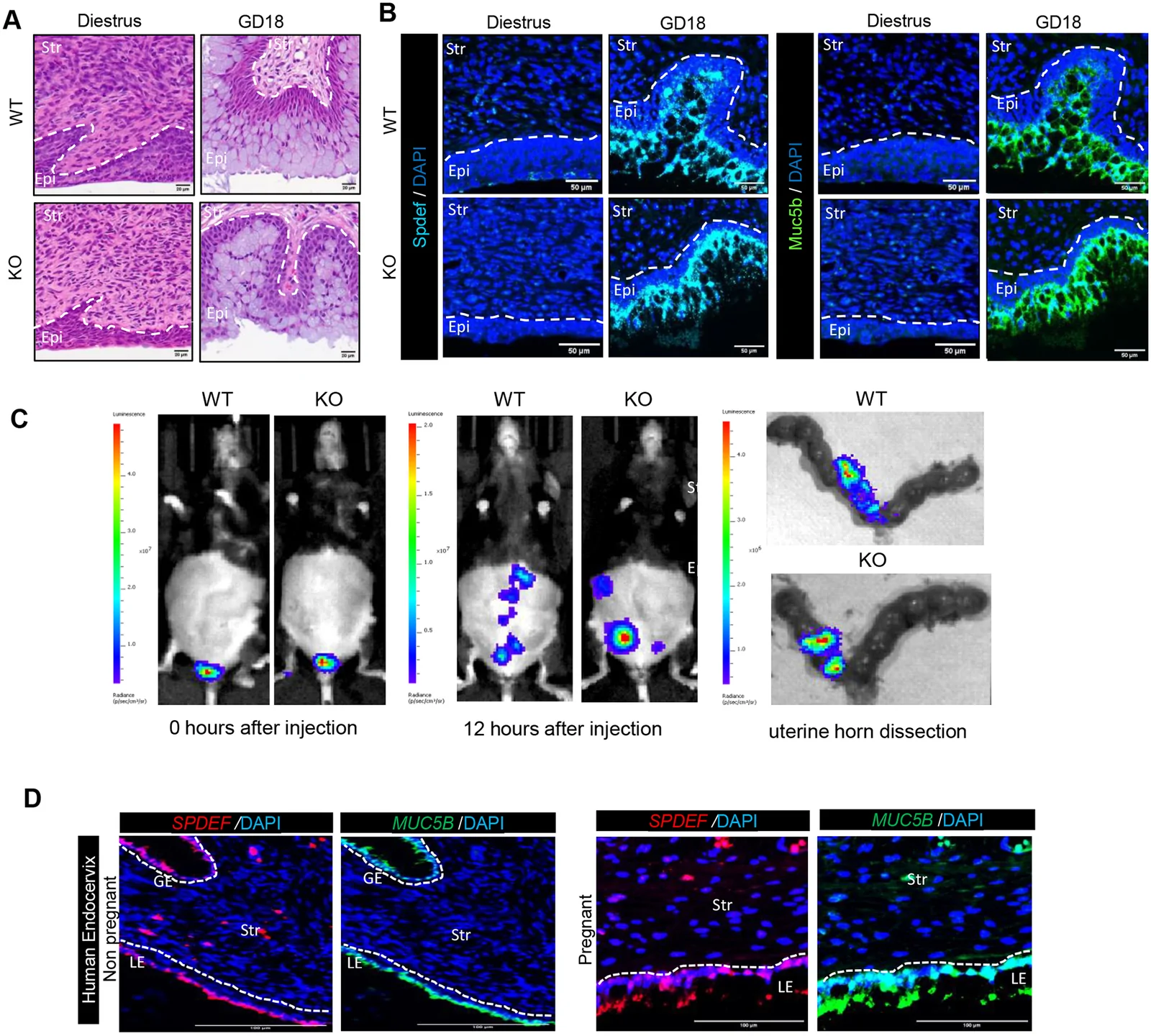
**OLFM4 expression is not required for cervical goblet cell differentiation or function.** (A) H&E staining of the endocervical canal in *Olfm4* WT and knockout (KO) mice during diestrus and at GD18. Images are representative of *n*=2 biological replicates per genotype and timepoint. (B) RNAscope staining for transcription factor Spdef (cyan) and Muc5b (green) in WT and KO endocervical tissues at non-pregnant (NP) diestrus and GD18. Both markers were detected in the same section, with each signal displayed in separate panels. Images are representative of *n*=2 biological replicates per genotype and timepoint. (C) Inoculation with bioluminescent, non-pathogenic *E. coli* (10^9^ CFU) at 0 and 12 h, followed by dissection of the uterine horns at 12 h post-inoculation in the WT and KO mice. Images are representative of *n*=3 biological replicates per timepoint. (D) RNA expression of *SPDEF* (red), *MUC5B* (green) and DAPI (blue) in human endocervix during NP and late pregnant states. Both markers were co-stained on the same section and are shown in separate panels. *n*=3 biological replicates per timepoint. Epi, epithelial region; GE, glandular epithelia; LE, luminal epithelia; Str, stromal region. Dashed lines show the boundary between epithelia and stroma. Scale bars: 20 μm (A); 50 μm (B); 100 μm (D).

Overall, these findings indicate that Olfm4 deficiency does not significantly affect cervical goblet cell differentiation, susceptibility to ascending *E. coli* infection, rates of PTB or proinflammatory responses to systemic LPS exposure. Consistent with this observation in mice, goblet cells in the human endocervix express canonical markers SPDEF and MUC5B (Gipson et al., 1999) (Fig. 5D) but do not express OLFM4 (Fig. S1B). Thus, while Olfm4-marked progenitors give rise to goblet cells, Olfm4 itself is dispensable for goblet cell differentiation and maintenance of the cervical mucosal barrier. The absence of a strong phenotype in Olfm4-deficient mice likely reflects redundancy with other secreted factors, as has been reported in the intestine and other mucosal tissues.

### Progesterone induces Olfm4^+^ progenitor differentiation into goblet cells

Progesterone is an established driver of secretory cell expansion and mucin synthesis, while estrogen promotes keratinocyte differentiation in the lower FRT (Ali et al., 2020; Arslan et al., 2015; Buchanan et al., 1998; Gipson, 2001; Greening et al., 2016; Kurita et al., 2001). We thus sought to evaluate the impact of progesterone on the proliferation and differentiation of the Olfm4^+^ luminal progenitor. To test this, female NP mice were ovariectomized (OVX) to deplete endogenous ovarian hormones and subsequently treated for 15 days with vehicle, estradiol (E2) or progesterone (P4). Cervical epithelial populations were then evaluated to assess the effects of each hormone (Fig. 6A).

**Fig. 6.**
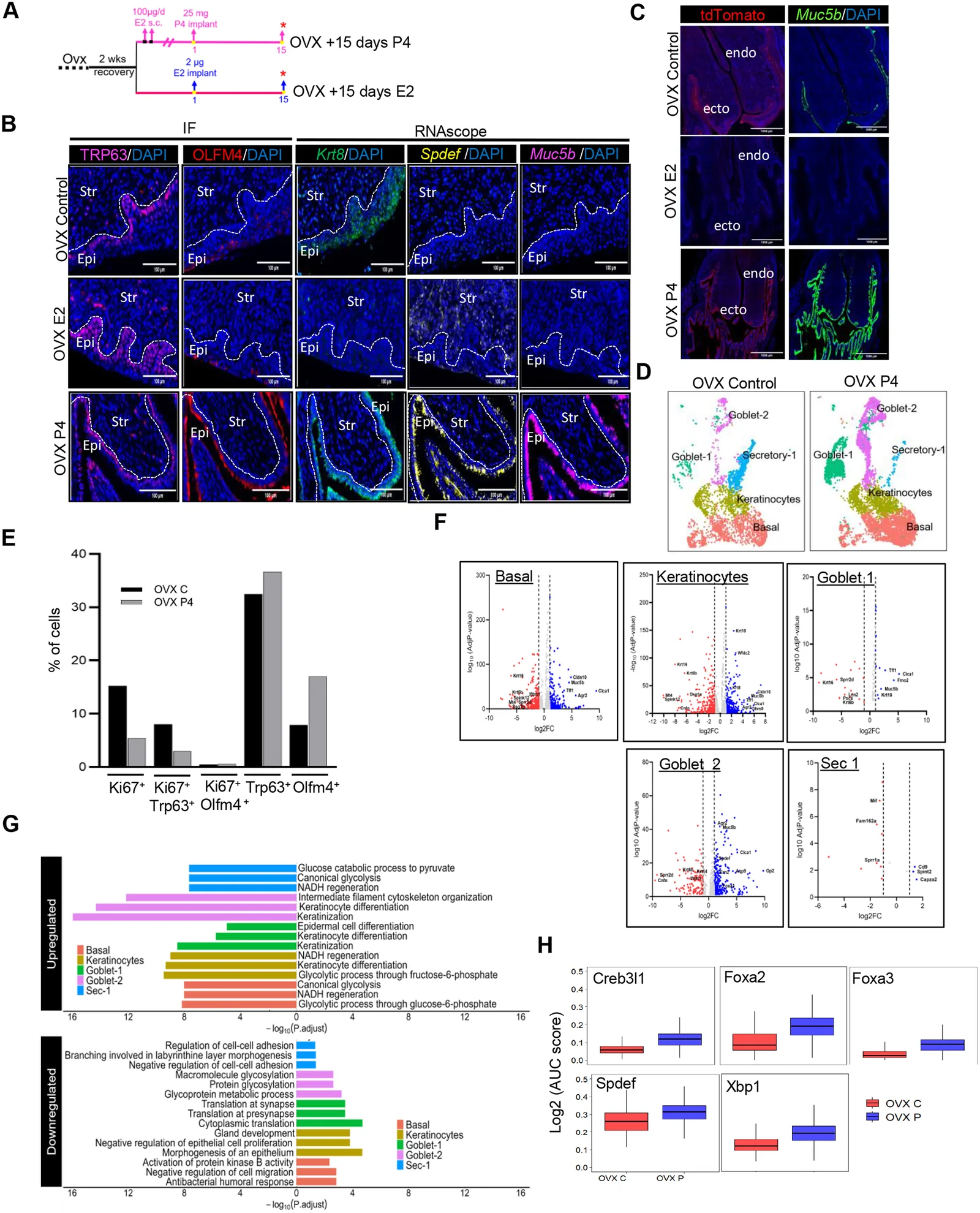
**Progesterone induces Olfm4^+^ progenitor differentiation into goblet cells.** (A) Schematic of hormonal treatment in ovariectomized (OVX) mice. (B) Immunofluorescence (IF) staining for TRP63 (basal; pink), OLFM4 (red), nuclei (blue). RNAscope staining for *Krt8* (green), *Spdef* (yellow) and *Muc5b* (pink). TRP63 and OLFM4 were detected on serial sections, whereas *Krt8*, *Spdef* and *Muc5b* were co-stained on the same section and displayed in separate panels. Epithelial (Epi) and stromal (Str) regions are indicated. Dashed lines show the boundary between epithelia and stroma. (C) RNAscope expression of tdTomato^+^ (red) and *Muc5b* (green) in Ai14Olfm4 lineage OVX control, OVX Estrogen (E2) and OVX Progesterone (P4). Both markers were co-stained on the same section and shown in separate panels. *n*=2 biological replicates per group. endo, endocervix; ecto, ectocervix. (D) UMAP visualization of single-cell cervical epithelial populations from OVX Control and OVX P4-treated mice. *n*=1 library per group; each library is comprised of a pool of nine cervices. (E) Percentage of proliferative epithelial cell subpopulations in OVX (black) and OVX P4 (gray) cervices. Bar graph shows the percentage of proliferating (Ki67^+^) and total Trp63^+^ basal cells and Olfm4^+^ luminal cells. As data were obtained from a single library per group, statistical analysis was not performed. (F) Volcano plots of differentially expressed genes (DEG) in OVX P4 versus OVX Control within each epithelial subtype (basal, keratinocyte, goblet-1, goblet-2, secretory-1) with selected upregulated and downregulated genes labeled. (G) Gene Ontology enrichment analysis of DEGs within each epithelial cluster indicating downregulated and upregulated pathways in the OVX P4 group. (H) SCENIC analysis of predicted transcription factor regulons induced in the Gob-2 epithelial cluster of OVX P4 samples. In the boxplots, the horizontal line within each box represents the median, and the lower and upper boundaries of the box represent the first and third quartiles (25th and 75th percentiles), respectively. Whiskers extend to the largest and smallest values within 1.5× the interquartile range (IQR) from the box boundaries. Scale bars: 100 μm.

Spatially distinct epithelial subpopulations were noted, with P4 treatment leading to increased Olfm4 protein expression and E2 treatment leading to increased expression of the keratinocyte markers *Dsg1a* and *Krt16* (Fig. 6B; Fig. S6A). TRP63 expression was consistently maintained across all treatment groups and predominantly localized to the basal layers. OLFM4^+^ cells were confined to the luminal layers, with a robust increase in OLFM4^+^ cells in the P4-treated but not E2-treated mice. In general, cells expressing *Krt8* transcripts overlapped with OLFM4^+^ luminal epithelium in the OVX control and both treatment groups. *Krt8* expression was lost under E2 treatment but induced by P4, with robust expression across multiple luminal layers. RNAscope staining for goblet cell markers *Spdef* and *Muc5b* demonstrated that the OLFM4^+^ cells induced by P4 treatment express these markers (Fig. 6B). The hormone treatment regimen as depicted in Fig. 6A was also utilized in NP Olfm4^Cre^ERT2; R26^tdTomato^ mice. The tdTomato^+^ cells persisted after ovariectomy and expanded with P4 but not E2 treatments (Fig. 6C).

We next sought to determine, at a single-cell resolution, the P4-driven transcriptomic changes in epithelial cell states that lead to goblet cell differentiation. Using the 10x Genomics platform, scRNA-seq libraries were generated from OVX control (OVX C) and OVX P4-treated cervices. After quality control filtering, canonical markers for epithelial, immune and stromal cells were used to annotate the dataset. In the current analysis, non-epithelial cells were computationally excluded from downstream analysis. The remaining epithelial cells were clustered into distinct epithelial subtypes using Seurat-predicted markers. Five epithelial clusters were identified in both OVX C and OVX P4-treated conditions: basal, keratinocyte, goblet-1 (Gob-1), goblet-2 (Gob-2) and secretory-1 (Sec-1) (Fig. 6D). As shown in Table S2, the OVX P4-treated group had a marked increase in the Gob-1 (20% versus 4%) and Gob-2 (23% versus 9%) clusters, a reduction in the Sec 1 (1% versus 24%) cluster, and only modest changes in the percentage of cells in the basal (37.5% versus 41%) and keratinocyte (18% versus 22%) clusters relative to the control group.

A comparison of proliferative *Ki67*^+^ populations between control and P4-treated groups indicated an overall decline in the percentage of proliferative cells with P4. While there was no change in the percentage of proliferating Olfm4 (*Ki67*^+^*Olfm4*^+^) cells, there was a marked reduction in the percentage of proliferating Trp63 (*Ki67*^+^*Trp63*^+^) cells in the P4-treated group. Thus, P4 does not induce proliferation of the Olfm4^+^ progenitor but does limit proliferation of the basal Trp63^+^ progenitor (Fig. 6E). The percentage of total *Olfm4*^+^ cells increased with P4 treatment relative to OVX C, consistent with the observed increase in goblet cells as seen in Fig. 6B. Gene markers that distinguish basal/keratinocyte cells were present in both control and P4-treated groups as seen in the UMAP visualization (Fig. S6B). Goblet clusters 1 and 2, which are enriched in the OVX P4 group, expressed markers of goblet cells (*Olfm4*, *Krt8*, *Spdef*, *Muc5b*, *Muc1*). The overall lower expression of these genes in Gob-1 compared to Gob-2 may indicate that Gob-1 cells are in an earlier stage of goblet cell differentiation. These two clusters are transcriptionally most similar to the NP/IL goblet clusters and pregnant goblet 1 cluster reported in Fig. 1B. Interestingly, Sec-1 was depleted in OVX P4 group-expressed *Muc1* and *Krt8*, but had little to no expression of goblet cell markers (*Olfm4*, *Spdef*, *Muc5*b). These studies support a role of P4 in goblet cell differentiation and are consistent with previous studies that identify P4 as a key regulator of secretory lineage commitment.

Differentially expressed genes in each epithelial subtype cluster in the OVX P4 and OVX C datasets were assessed (Fig. 6F). Upregulated genes in Gob-1 and Gob-2 clusters included goblet cell markers (*Spdef*, *Muc5b*, *Clca1*), while downregulated genes were markers of squamous keratinizing cells (*Krt16*, *Krt6*, *Cnfn*). Although the number of Sec-1 cells declined in the OVX P4 group, relatively few genes were differentially expressed in this cluster compared to the OVX C group. Interestingly, in basal and keratinocyte clusters, P4 significantly upregulated genes associated with secretory cells (*Cldn10*, *Muc5b*, *Tff1*, *Clca1*, *Agr2*), while downregulating genes associated with keratinocyte cells (*Krt16*, *Krt6b*, *Spink12*, *Mt4*, *Sprr2d*, *Dsg1a*, *Ifitm1*, *Cnfn*) (Fig. 6F). Gene Ontology (GO) pathway analysis (Fig. 6G) revealed progesterone-induced upregulation of glycoprotein metabolic processes and downregulation of keratinization pathways across subtypes. This was further supported by *in situ* RNA hybridization for keratinocyte genes *Dsg1a* and *Krt16* (Fig. S6A). Both genes were expressed at low levels in the OVX C group, induced in OVX E2- and suppressed in the OVX P4-treated groups. Basal cells had reduced cell migration, while upregulated pathways in keratinocytes and goblet/secretory cells were consistent with increased secretory cell function (Fig. 6G). Progesterone-induced transcription factor (TF) activity analysis using SCENIC identified numerous TFs demonstrated to regulate goblet or secretory cell development or function, specifically enriched in Gob-1 and Gob-2 clusters (Fig. 6H). Common to all clusters and consistent with known progesterone receptor (PR) functions were TFs necessary for suppression of pro-inflammatory responses (Fig. S6C). In addition, TRIM28, previously described as a binding partner for PR necessary for uterine function (Li et al., 2023), was among the top 12 TFs with high activity in the luminal cluster (Fig. S6C). Collectively, these findings support the role of progesterone in reorganizing cervical epithelial cell states. Specifically, progesterone directs Olfm4^+^ progenitors towards goblet cell differentiation, activating a transcriptional program that promotes secretory function while simultaneously downregulating the expansion and/or differentiation of Trp63^+^ progenitors into stratified squamous subtypes.

## DISCUSSION

The findings of this study demonstrate that the adult mouse lower FRT epithelium is maintained by two progenitor pools: a columnar Olfm4^+^ progenitor that gives rise to goblet cells, and a Trp63^+^ basal progenitor that gives rise to stratified squamous cells (Fig. 7A). Differentiation of these specialized lineages is regulated, in part, by the steroid hormone environment. Specifically, progesterone promotes transcriptional programs that drive goblet cell differentiation from Olfm4^+^ progenitors, while limiting proliferation and differentiation of Trp63^+^ basal progenitors. In the adult, cell state changes in secretory cell clusters distinguish goblet cells clusters in early stages of differentiation from mature goblet cell clusters (Fig. 7B). The UMAP in Fig. 7B has been updated from Fig. 1B to assign clusters as early goblet versus mature goblet.

**Fig. 7.**
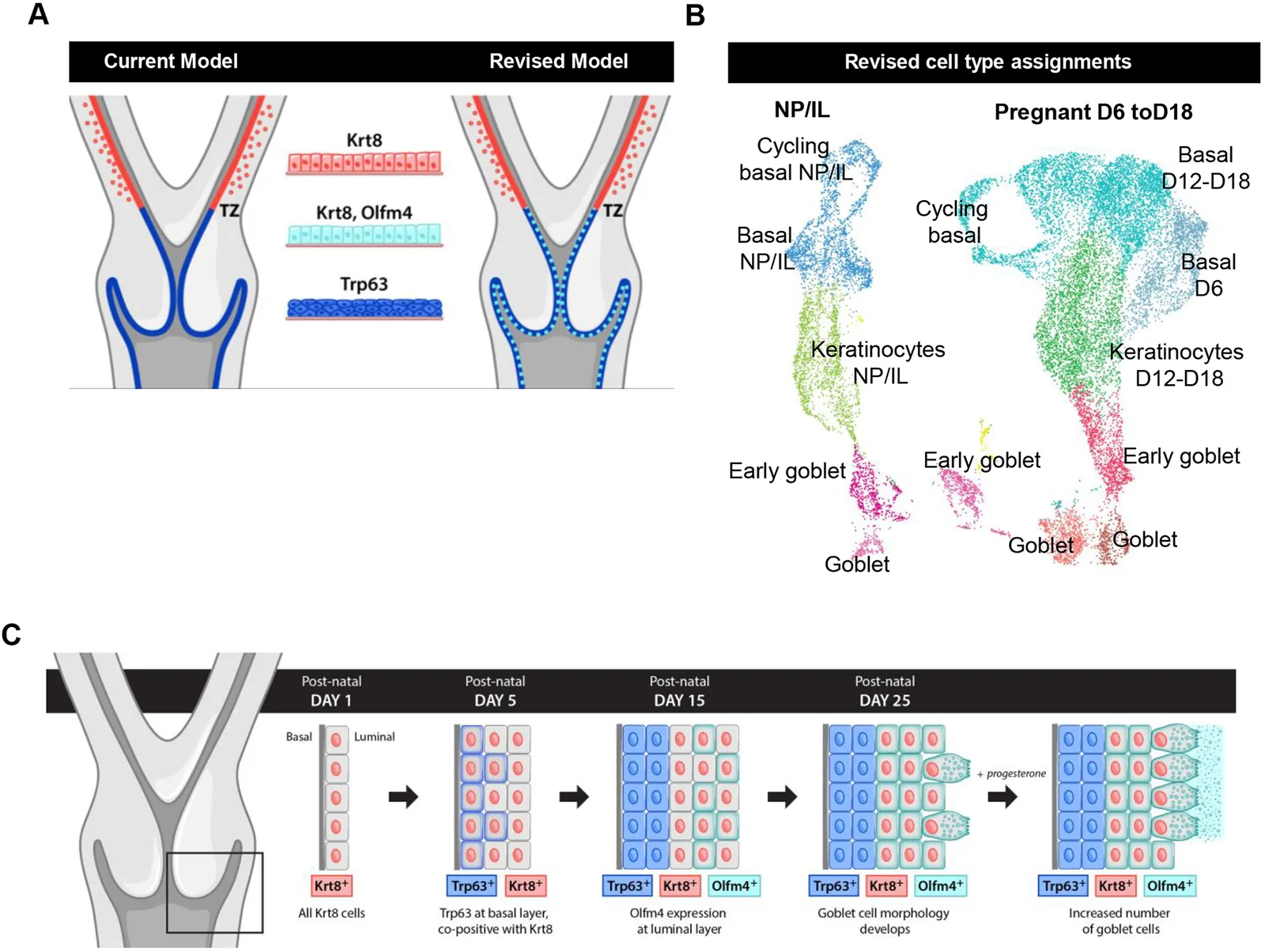
**Two epithelial progenitors delineate the postnatal mouse cervix and vagina and revise the single-progenitor model.** (A) Comparison of the current and revised models of cervicovaginal epithelial lineage. In the current model, the uterine epithelium is lined by Krt8^+^ (red) columnar cells, while the cervix and vagina are lined by a Trp63^+^ (blue) stratified squamous epithelium derived from a single Trp63^+^ basal progenitor. In the revised model, the cervicovaginal epithelium is maintained by two progenitors: a Trp63^+^ (blue) basal progenitor that gives rise to stratified squamous cells, and a Krt8^+^Olfm4^+^ (cyan) progenitor that gives rise to goblet cells at the luminal surface. TZ, transformation zone. (B) UMAP of mouse cervical epithelium across non-pregnant, in-labor and pregnant (GD6-GD18) timepoints, annotated by goblet cell differentiation state. The secretory clusters shown in Fig. 1B are here annotated as early goblet or goblet. Basal, cycling basal and keratinocyte populations are labeled as in Fig. 1B. (C) Schematic of epithelial progenitor delineation during postnatal development of the mouse cervix and vagina. At PND1, the epithelium is uniformly Krt8^+^ (red). By PND5, the multilayered epithelium remains Krt8^+^, with basal cells co-expressing Trp63 (blue). By PND15, Olfm4 (cyan) expression is initiated in a Krt8^+^ luminal subset, delineating an Olfm4^+^ luminal progenitor distinct from the Trp63^+^ basal progenitor. By PND25, goblet cell morphology is established, and progesterone promotes an increase in goblet cell number. The boxed region in the whole-tract schematic (left) indicates the area depicted.

The FRT is lined by secretory epithelia with regionally distinct functions. During pregnancy, secretions from the uterine epithelia support implantation and fetal development, while cervical epithelia secretions form a mucosal barrier that protects against ascending infections to ensure a term pregnancy (Cunha et al., 2017; Kelleher et al., 2019; Lacroix et al., 2020; Wira et al., 2010). This study highlights lineage distinctions in the lower FRT compared to the uterus that are crucial for the formation of a mucosal barrier. In particular, the transcriptional specification of goblet cell progenitors is established by PND15 in the cervix and vagina (Fig. 7B). In the gastrointestinal and respiratory tracts, goblet cells arise from columnar progenitors (Gustafsson and Hansson, 2025). Similarly, in the human endocervix, goblet cells arise from columnar epithelium and are present in the endocervix before and during pregnancy (Fig. 5D) (Madhukaran et al., 2025; Ueda et al., 2022). Here, we extend this paradigm to the mouse cervix, showing that goblet cells have a columnar origin and are derived from a luminal progenitor pool that is distinct from the basal progenitor population present in the cervix and vagina.

Previous studies support the existence of a single progenitor that maintains the adult mouse vaginal and cervical epithelium. Lineage-tracing studies in adult OVX mice identified a basal Axin2^+^ stem cell capable of regenerating all layers of the vaginal keratinized epithelium in response to E2 exposure (Ali et al., 2020). Similarly, clonal expansion studies of Trp63^+^ basal progenitor cells from mouse or human cervix generated both keratinized epithelia (KRT10^+^ or KRT13^+^) and secretory cells (MUC1^+^) (Zhao et al., 2021). From these studies, the authors concluded that basal progenitors could give rise to both squamous and secretory cells. However, these studies did not demonstrate that goblet cells originate from basal progenitors, nor was it appreciated that goblet cells are present in the mouse vagina and ectocervix.

Collectively, our findings revise the current model in which columnar epithelium lining of the mouse lower reproductive tract at birth undergoes postnatal transdifferentiation to squamous epithelium induced by the expression of the transcription factor Trp63 on PND5-PND8 (Kurita, 2010; Kurita and Cunha, 2001; Kurita et al., 2005; Terakawa et al., 2016). We propose refinement to this model during postnatal development (Fig. 7C), in which a portion of Krt8^+^ cells acquire Trp63 expression and transition to squamous epithelium, while a subset remains Krt8^+^Trp63^−^ and subsequently acquire Olfm4 expression. This Olfm4 population serves as the unipotent progenitor pool for adult goblet cells in the cervix and vagina. Thus, the cervical and vaginal epithelium in mice harbors both basal Trp63^+^ and columnar Olfm4^+^ progenitors.

While Olfm4 is not required for goblet cell differentiation or barrier function in mice or humans, Olfm4 serving as a progenitor marker in the murine cervix parallels findings in other epithelial tissues such as the urethral tube and prostate epithelium, where Olfm4 marks progenitor-like cells (Li et al., 2020). Olfm4 expression occurs in a spatial gradient, with Olfm4^+^ progenitors first appearing in vaginal epithelia and later extending to the ectocervix and endocervix between PND15 and PND25. The possibility that Olfm4 is a conserved marker for progenitor secretory cells during FRT development in the human is supported by the expression of Olfm4 in lower vaginal epithelium and urethral epithelia in single-cell transcriptomic datasets from females at postconception weeks 11-21 (Lorenzi et al., 2025). In addition, the transcriptional regulator of goblet cells, SPDEF is also expressed in this dataset in the fallopian lower uterus/cervix and vaginal epithelia.

Though we observed distinct populations of epithelial layers expressing Trp63 or Krt8 in the postnatal period, we cannot rule out the possibility that a small pool of bipotent progenitors persists to adulthood in the lower FRT and gives rise to both the basal Trp63^+^ and luminal Olfm4^+^ progenitor pools in specific physiological periods (e.g. pregnancy) or with tissue injury or disease. This possibility is supported by the presence of minor populations of Trp63 and Krt8 co-positive cells in cervices of mice and humans. In our human studies, cells co-expressing squamous (TRP63) and columnar marker (KRT8), while not uniform across all patients, resemble ‘reserve cells’ previously described in the human cervix (Martens et al., 2007, 2009) and may reflect a transitional, plastic cell state.

Overall, this study advances our understanding of lower tract epithelial differentiation during the postnatal developmental period in mice and reveals previously unrecognized species similarities between mice and humans in which goblet cell and keratinized cell subtypes are derived from columnar and basal progenitors, respectively. In humans, columnar progenitors are restricted to the endocervix, while basal squamous progenitors are confined to the ectocervix, potentially explaining the spatial restriction of goblet cells in humans compared with their broader distribution in mice.

This framework provides insight into epithelial plasticity required to maintain barrier homeostasis in the cycling and pregnant cervix. The rapid pivot in epithelial subtypes necessary to maintain tissue homeostasis raises questions as to the cell intrinsic and extrinsic signals that regulate this process. Steroid hormones and growth factors are key regulators of reproductive tract epithelial proliferation, differentiation and maintenance of cell-fate (Chumduri et al., 2021; Kurita et al., 2000; Mehta et al., 2016; Rizo et al., 2023; Yin et al., 2025). Previous studies have demonstrated that region-specific signaling cues from the underlying stroma drive epithelial differentiation during postnatal development and in the adult to maintain cell-fate specification (Cunha, 1976; Kurita et al., 2001). In addition, exogenous exposure of hormones, for example diethylstilbestrol, during FRT development in mice or humans perturbs epithelial differentiation in the adult FRT and contributes to risk of adult-onset cancers of the FRT (Kurita et al., 2004; Padilla-Banks et al., 2023). Our findings suggest that hormone signaling fine-tunes the balance between basal and columnar cervical progenitor populations. Future studies will aim to define the intrinsic and extrinsic signals governing this balance and to determine how goblet cell function contributes to mucosal defense and pregnancy outcomes.

### Limitations of the study

Our study has several limitations. We refer to the basal progenitor as a Trp63^+^ basal progenitor, based on previous *Trp63* gene deletion studies (Kurita et al., 2005; Romano et al., 2012). As isoform-specific Trp63 Cre reporters are not available, we utilized a Krt5Cre^ERT2^; R26^tdTomato^ reporter, since Krt5 is considered a specific marker of stratified squamous cells. Unexpectedly, we observed tdTomato^+^ cells in the uterine luminal and glandular epithelia and in all layers of the cervical epithelium. The Krt5 lineage result is supported by low expression of Krt5 protein in the mouse uterus and high expression in the cervix (Fig. S7). Consistent with our observation, the presence of non-basal Krt5^+^ endometrial subpopulations in human scRNA-seq datasets has been recently described (Nikolakopoulou et al., 2026). Future lineage-tracing studies using isoform-specific Trp63 reporter Cres are necessary to solidify lineage specificity of a Trp63^+^ basal progenitor. Finally, human tissue analyses were limited to a small number of NP and late-pregnant samples, restricting the ability to capture dynamic changes across the menstrual cycle and gestation.

## MATERIALS AND METHODS

### Reagents and antibodies

The following chemicals were used: Tmx (Sigma-Aldrich, #T5648; CAS#10540-29-1), progesterone pellets (Innovative Research of America, #P-131), estrogen pellets (Innovative Research of America, #E-121). The following primary antibodies were used: rabbit anti-P63 (D9L7L) (Cell Signaling Technology, #39692S, 1:200; RRID:AB_2799159), mouse purified anti-Cytokeratin 8 (BioLegend, #904804, 1:200; RRID:AB_2616821), rabbit anti-Olfm4 mAb (D6Y5A) (Cell Signaling Technology, #39141, 1:200; RRID:AB_2650511), goat tdTomato polyclonal antibody (My BioSource, #MBS448092, 1:100; RRID:AB_2827808), rat anti-Ki67 mAb (SolA15) (Thermo Fisher Scientific, #14-5698-82, 1:200; RRID:AB_10854564), mouse anti-Keratin 8 (Abcam, #Ab9023, 1:200; RRID:AB_306948), anti-Muc5AC (Abcam, #ab3649, 1:400; RRID:AB_2146844), Ghost Viability Dye 510 (Tonbo Biosciences, #SKU-13-0870-T100, 1:1500), rat anti-mouse CD16/CD32 (BD Biosciences, #553141, 1:200; RRID:AB_394656), anti-EpCAM (Abcam, #ab71916, 0.5:200; RRID:AB_1603782), E-Cadherin (Cell Signaling Technology, #3195, 1:200; RRID:AB_2291471), goat anti-rabbit IgG Alexa Fluor 488 (Thermo Fisher Scientific, #A11008, 1:200; RRID:AB_143165), donkey anti-goat IgG Alexa Fluor 647 (Thermo Fisher Scientific, #A21447, 1:200; RRID:AB_141844), goat anti-rabbit IgG Alexa Fluor 594 (Thermo Fisher Scientific, #A-11012, 1:200; RRID:AB_2534079), goat anti-mouse IgG Alexa Fluor 488 (Thermo Fisher Scientific, #A-11001, 1:200; RRID:AB_2534069), goat anti-rat IgG Alexa Fluor 488 (Thermo Fisher Scientific, #A-11006, 1:200; RRID:AB_2534074), donkey anti-goat IgG Alexa Fluor 594 (Thermo Fisher Scientific, #A-11058, 1:200; RRID:AB_2534105). The following bacterial strains were used: *E. coli* O55:K59(B5):H- [ATCC, #12014; strain CDC 5624-50 (NCTC 9701)], *E. coli* K12 nonpathogenic strain [gift from Dr Michael House (Tufts University), pAKlux2.1 plasmid]. The following RNAscope probes were used: mouse Spdef probe (Advanced Cell Diagnostics, #544421), mouse Muc5b probe (Advanced Cell Diagnostics, #471991), mouse Muc1 probe (Advanced Cell Diagnostics, #421871), mouse Olfm4 probe (Advanced Cell Diagnostics, #311831), mouse Dsg1a (Advanced Cell Diagnostics, #842861), mouse Krt16 (Advanced Cell Diagnostics, #1153201), human SPDEF (Advanced Cell Diagnostics, #543151), human MUC5B (Advanced Cell Diagnostics, #449881).

### Animals

All animal experiments and protocols were approved by the Institutional Animal Care and Use Committee at UT Southwestern. All mice were housed in a controlled environment with regulated temperature and a 12 h light/12 h dark cycle, having free access to water and food in the animal facility. In this study, we used the WT C57BL/6;129 (B6129F1) mouse strain, along with Olfm4-IRES-eGFPCreERT2, Krt8-Cre/ERT2(17Blpn/J) (strain 017947), and Rosa26tdTomato (strain 007914) mice. The Olfm4-IRES-eGFPCreERT2 mice were generated in the lab of Dr Hans Clevers (Hubrecht Institute, Utrecht, The Netherlands) and kindly provided by Dr Linda Samuelson at the University of Michigan with permission from Dr Clevers. The Rosa26tdTomato mice were provided by Dr Chun-Li Zhang at UT Southwestern Medical Center. The Krt8-Cre/ERT2(17Blpn/J) mice and B6N.129S6(Cg)-Krt5tm1.1(cre/ERT2)Blh/J (stock #029155) were sourced from Jackson Laboratories. The Olfm4-IRES-eGFPCreERT2 and Krt8-Cre/ERT2(17Blpn/J) lines have been previously described (Schuijers et al., 2014; Van Keymeulen et al., 2011). Olfm4^−/−^ mice and Olfm4 WT mice were generated previously (Liu et al., 2010) and provided by Drs Wenli Liu and Griffin Rogers at the National Heart, Lung, and Blood Institute.

### Microbe strain

Two *E. coli* strains were used in this study. Live *E. coli* (serotype O55; ATCC) (20 μl bacteria equal to 10^7^ CFU) was inoculated into vagina on GD15, and mice were observed for 72 h for signs of PTB as per a previous study protocol (Akgul et al., 2014). The bacteria were cultured aerobically at 37°C in sterile, nonselective Luria-Bertani (LB) broth overnight with shaking at 200 rpm. Cultures were harvested by centrifugation (4000 ***g*** for 5 min) and re-suspended at the desired concentration with PBS. The correct cell concentration for vaginal inoculation was calculated by measuring an OD600 against the standard growth curve for that bacterial strain. To confirm the dose of bacteria given, an aliquot of the bacterial culture was serially diluted 1:100 to an approximate bacterial concentration of 500 CFU/ml. A 100-μl aliquot was plated under sterile conditions, and colonies were counted after overnight incubation at 37°C to confirm the accuracy of the CFU. Delivery within 72 h of inoculation is considered PTB, whereas delivery on or after GD19 is considered a term birth.

Nonpathogenic *E. coli* K12 used for bioluminescent imaging was constructed according to a previous protocol and sent as a gift from our collaborator Dr Michael House (Zhang et al., 2024). Bacterial inoculate was prepared by growing *E. coli* K12-lux in 30 ml LB broth containing 50 μg/ml ampicillin overnight at 37°C with shaking at 200 rpm. Cultures were harvested by centrifugation (5000 ***g*** for 5 min). The correct cell concentration for vaginal inoculation was calculated by measuring an OD600 against the standard growth curve for that bacterial strain. After centrifugation, the bacterial pellet was resuspended in a predetermined volume of PBS and introduced to the mice. Ampicillin resistant *E. coli* colonies were checked for light emission at peak emission of 490 nm (IVIS Spectrum Imaging System). Vaginal inoculation of 20 μl of 1×10^9^ CFU *E. Coli* was performed at GD15 followed by whole-body bioluminescence imaging at 0 h and 12 h post inoculation. At 12 h, the mice were euthanized and dissected. Fluorescence was detected with the IVIS Spectrum Imaging System in the amniotic sac, placenta, uterus and pups.

### Tissue collection

All mice used in NP and pregnant studies were 2-6 months old and nulliparous. Adult mice used in this study were all females. The stage of the NP estrus cycle in females was determined by vaginal cytology, based on the presence, absence and proportions of cornified epithelial cells, nucleated epithelial cells and leukocytes (Byers et al., 2012). Timed pregnancies were generated by pairing females with males overnight (16.00 h to 08.00 h), and the following morning females were checked for the presence of a vaginal plug. The day that a plug was detected was designated as GD0, with birth typically occurring on GD19. NP (estrus, metestrus, diestrus), pregnant (GD6, GD12, GD15, GD18, GD19) and IL samples were collected for analysis. The Olfm4-Cre^ERT2^;Rosa26^tdTomato^ line delivered on GD19-GD20. IL samples were collected on GD19 or GD20 after visual confirmation of the delivery of one or two pups. For tissue collection in the postnatal period in WT mice, cervical tissues were harvested at PND1, PND5, PND15 and PND25 following sex determination of the pups, as previously described (Papaioannou and Behringer, 2024).

### Lineage-tracing studies

The Olfm4 IRES-eGFPCre^ERT2^ and Krt8 Cre/^ERT2^(17Blpn/J) mice were crossed with Rosa26^tdTomato^ mice to generate Olfm4-IRES-eGFPCre^ERT2^; Rosa26^tdTomato^ and Krt8Cre/^ERT2^; Rosa26^tdTomato^ reporter lines. Genotyping was performed via PCR on genomic DNA extracted from tail samples (Jacquot et al., 2019). For lineage tracing, reporter females were paired overnight with Rosa26-tdTomato males, and timed pregnancies and IL sample collection were performed as described above. To induce Cre-mediated recombination, adult females heterozygous for Olfm4-IRES-eGFPCre^ERT2^;Rosa26^tdTomato^ or Krt8 Cre/^ERT2^;Rosa26^tdTomato^ were injected intraperitoneally with a single dose of Tmx (Sigma-Aldrich, T5648) dissolved in corn oil (1 mg in 100 μl) at 8 weeks of age. For experiments conducted in OVX Olfm4-IRES-eGFPCre^ERT2^; Rosa26^tdTomato^ females, mice received two consecutive daily injections of Tmx (1 mg/day), then were rested for 1 week before ovariectomy as described below in the ‘Ovariectomized studies’ section.

### Lineage tracing performed at both short- and long-term intervals

For short-term induction, Tmx was administered during estrus or diestrus, or on GD11, and tissues were collected at estrus, diestrus or GD12, respectively. For long-term induction, Tmx was administered during NP stages, and mice rested for 1 week before timed mating. Tissues were collected during the subsequent reproductive cycle at estrus (postpartum), diestrus and GD15 of a second pregnancy.

### Ovariectomized studies

C57BL/6;129 female mice (7-8 weeks old) were OVX and allowed a 2-week recovery period before treatment. Females were then separated and assigned to one of the three groups: Control, estrogen (E2) and progesterone (P4). Control group: OVX-control mice received no treatment during the 15-day experimental period. Estrogen (E2) group: OVX mice were implanted with a 0.05 mg 21-day release pellet (Innovative Research of America) providing an estimated daily release of 2 μg. Progesterone (P4) group: OVX mice received a subcutaneous injection of E2 (100 μg/day in 100 μl of corn oil) for two consecutive days to induce progesterone receptor expression, followed by implantation of a 25 mg P4 21-day release pellet (Innovative Research of America), providing an estimate daily release of 1 mg. Tissues from all three groups were collected for immunostaining after 15 days of treatment. For single-cell RNA-seq library preparation, cervical cells were isolated from OVX control and OVX P4 groups as described previously (Cooley et al., 2023; Madhukaran et al., 2023).

### Mouse tissue collection and histological evaluation

Cervices and uteri were isolated by dissection at the uterocervical junction. Cervix, vagina and uterine horns were collected as a single piece and oriented for longitudinal sectioning, with the cervical canal visible throughout each section. All tissues were fixed in 4% paraformaldehyde (Sigma-Aldrich) in 1× PBS for 24 h at 4°C and then transferred to 1× PBS. Tissue comprising the cervix, vagina and part of the uterine horns was embedded in paraffin and 5 μm thick longitudinal sections were cut. Hematoxylin and Eosin (H&E) and Alcian blue-PAS staining were performed by the Histo-Pathology Core at UT Southwestern Medical Center.

### Immunohistochemistry and immunofluorescence staining

Immunohistochemistry (IHC) and immunofluorescence (IF) were performed according to standard protocols (Cooley et al., 2023) . IHC/IF was performed on deparaffinized and rehydrated 5 μm sections following heat-induced antigen retrieval in sodium citrate buffer (10 mM, pH 6.0) at ∼95-100°C for 20 min. Staining was repeated on at least three sections from three independent animals (biological replicates) and representative immunostainings are shown.

### *In situ* mRNA hybridization

RNAscope was performed using RNAscope^®^ Multiplex Fluorescent Reagent Kit V2 (Advanced Cell Diagnostics) as previously described (Cooley et al., 2023). RNAscope was repeated on tissue sections collected from one to three biological replicates and representative immunostainings are shown.

### Human tissues

All experimental procedures involving human tissues were approved by the Institutional Review Board at the University of Texas Southwestern Medical Center (IRB #112010-047) and written informed consent was obtained from all participants. NP (premenopausal) and pregnant (34-36 weeks gestation) cervical tissue was obtained from the UTSW Department of Obstetrics and Gynecology Tissue Biorepository. Pregnant ectocervical and endocervical samples were collected at the time of hysterectomy for complications associated with placenta accreta spectrum. Inclusion criteria were 18 years or older, English/Spanish speaking, consent to study. Exclusion criteria were placenta previa, placental invasion into cervical tissue, abnormal high risk pap test/HPV testing. Transmural sections (10×10 mm) were wrapped in Kimwipes to preserve endocervical canal epithelial cells and fixed in 4% paraformaldehyde for 24 h, followed by transfer to PBS. Longitudinal sections (5 μm thick) of endocervix or ectocervix were then prepared. H&E staining was performed by the Histo-Pathology Core at UTSW.

### Systemic LPS-induced PTB

Preterm labor was induced by intraperitoneal injection of 100 μl LPS (serotype O55, Sigma-Aldrich) at 5 mg/kg concentration on GD15 in WT and OLFM4-deficient mice. Animals were either observed for PTB or euthanized 6 h after injection (before onset of PTB) for tissue collection.

### Image analysis

The whole-slide immunohistochemistry samples were scanned using the Hamamatsu Nanozoomer S60 and viewed using NDP.view2 software, as previously described (Cooley et al., 2023). Immunofluorescence and RNAscope images of longitudinal cervical sections were acquired using a Zeiss Axioscan Z1 digital slide scanner (ZEN 3.0 software) at 20× magnification. Higher-resolution images were acquired on a Zeiss LSM 880 inverted laser-scanning confocal microscope equipped with a two-photon module. To ensure consistency across timepoints, all fluorescent images, including RNAscope with TSA dyes, were captured under identical detector gain and laser power settings using the appropriate filter configurations. Standard excitation and emission settings were: DAPI (358/461 nm), FITC/TSA 520 (494/525 nm), Cy3/TSA 570 (550/570 nm), Cy5/TSA 650 (650/673 nm) and Cy5.5/TSA 690 (676/696 nm). For temporal comparisons, optimal imaging conditions determined at the lowest-signal timepoint were applied to all subsequent timepoints, and images were linearly rescaled per channel. Scale bars were added using ImageJ.

### Cell isolation for tdTomato sorting from Ai14Olfm4 lineage mice

Olfm4-IRES-eGFPCreERT2;Rosa26tdTomato females (*n*=3; two independent experiments) were administered two consecutive intraperitoneal doses of Tmx (1 mg each) during the NP stage. Cervical tissues were harvested at diestrus, minced (∼1 mm^3^) and digested overnight at 4°C on a rotating platform in Dispase 5 U/ml (Stemcell Technologies, #7913) solution. Digests were diluted in ice-cold PBS or Advanced DMEM/F12 supplemented with GlutaMax, HEPES and Penicillin/Streptomycin, filtered through a 40 μm strainer and centrifuged (1500 rpm, 5 min, 4°C). Pellets were resuspended in ice-cold 1× PBS(∼100-200 μl) for flow cytometry.

### Cell staining for flow cytometry and analysis

Single-cell suspensions were divided into experimental and control tubes. Controls included: (1) unstained WT NP cells (autofluorescence baseline), (2) viability dye only, (3) single-color controls for EpCAM and tdTomato, and (4) secondary-antibody-only controls (anti-rabbit 488 and anti-goat 647). Experimental samples were incubated with Ghost Viability Dye 510 (Tonbo Biosciences; Cytek SKU-13-0870-T100, 1:1500), followed by Fc receptor blockade with BD Pharmingen™ anti-CD16/CD32 (mouse BD Fc Block™, #553141, 1:200). Because tdTomato reporter expression in the NP diestrus cervix was low and not reliably detected by native fluorescence, antibody-based detection was used to enhance signal. To ensure consistent quantification across reproductive stages, the same antibody-based detection was applied to all flow cytometry samples, including pregnancy, where native tdTomato is more abundant. Cells were incubated on ice for 30 min with rabbit anti-EpCAM (Abcam, ab71916, 1:200) and goat anti-tdTomato (MyBiosource, MBS448092, 1:200 for flow sorting). After washing, cells were incubated on ice for 30 min with Alexa Fluor 488 conjugated goat anti-rabbit IgG (Invitrogen, A11008, 1:200) and Alexa Fluor 647 conjugated donkey anti-goat IgG (Invitrogen, A21447, 1:200). Final washes were performed, and cells were resuspended in Advanced DMEM/F12 supplemented with GlutaMax, HEPES and Penicillin/Streptomycin and passed through 40 μm-filtered fluorescence-activated cell sorting (FACS) tubes. Flow cytometry and sorting were performed using an Aurora CS. Live epithelial cells were gated by EpCAM expression and viability dye exclusion. In Ai14Olfm4 samples, luminal progenitors were defined as EpCAM^+^ tdTomato^+^, while basal progenitors were defined as EpCAM^+^ tdTomato^−^. Data were analyzed using FlowJo v10.9.0.

### Organoid culture

Flow-sorted populations (EpCAM^+^ tdTomato^+^ luminal progenitors and EpCAM^+^ tdTomato^−^ basal progenitors) were embedded separately in growth factor reduced basement membrane extract (BME; Cultrex) and cultured in cervical organoid expansion medium as previously described (Lõhmussaar et al., 2021). Organoids were maintained at 37°C in 5% CO_2_, with medium replaced every 3 days. After 10-12 days, organoids were imaged by confocal microscopy to verify tdTomato^+^ luminal cell contribution. In organoids, tdTomato^+^ cells are clonally expanded, yielding native fluorescence readily detectable by confocal microscopy without antibody amplification. Organoids from three biological replicates were maintained for at least three passages over ∼5 weeks (passages every 12 days). Organoid formation assays were conducted for each biological replicate using three technical (wells) replicates (5000 cells/25 μl drops; 2 drops per well) and the data are presented as the mean±s.e.m. Statistical differences between two groups were determined with Welch's t-test (GraphPad Prism 10.5), and statistical significance was defined as *P*<0.05.

### RNA isolation and quantitative real-time PCR

Total RNA was extracted from tissues using RNA-STAT 60 (Tel-Test) and treated with DNase I (DNA-Free, Ambion). cDNA was synthesized from 1 μg of total RNA in a 20 μl reaction using the iScript Reverse Transcription Supermix (Bio-Rad). Quantitative real-time PCR was performed with SYBR Green chemistry on a CFX384 Touch Real-Time PCR Detection System (Bio-Rad). Reactions were run in triplicate with 7.6 ng cDNA per well. Gene expression levels were normalized to the housekeeping gene *36B4* (also known as *Rplp0*) , and relative expression was calculated using the 2^−^ΔΔCt method. Predesigned SYBR Green primers for mouse inflammatory genes (*Tnf*, *Il6*, *Il1b* and *Cxcl2*) were purchased from Sigma-Aldrich. The following primers were used for qPCR: *Il6* Forward (5′-TCGTGGAAATGAGAAAAGAGTTG-3′), *Il6* Reverse (5′-AGTGCATCATCGTTGTTCATACA-3′), *Cxcl2* Forward (5′-GAACATCCAGAGCTTGAGTGTGA-3′), *Cxcl2* Reverse (5′-CCTTGAGAGTGGCTATGACTTCTGT-3′), *Il1b* Forward (5′-GCCCATCCTCTGTGACTCATG-3′), *Il1b* Reverse (5′-AGCCTGTAGTGCAGCTGTCTAATG-3′), *Tnf* Forward (5′-CTGAGGTCAATCTGCCCAAGTAC-3′), *Tnf* Reverse (5′-CTTCACAGAGCAATGACTCCAAAG-3′).

### ScRNA-seq of OVX samples and analysis

Reads were aligned to the mouse genome (mm10) using Cell Ranger v3.0.2. Downstream processing was performed with Seurat v5 (Hao et al., 2024) following the standard workflow (https://satijalab.org/seurat/articles/get_started_v5_new). Cells with >300 detected genes and <10% mitochondrial transcripts were retained; genes detected in at least ten cells were included. After filtering, 4148 and 7659 cells were retained for the OVX control and OVX P4 samples, respectively. Normalization across conditions was performed using SCTransform, and datasets were integrated with the Harmony algorithm via the Seurat ‘integrate layers’ function without regression of variables. The top 30 principal components were used for clustering with a resolution of 0.8, yielding 21 clusters. Cell types were assigned based on canonical marker genes (Cooley et al., 2023), resulting in five major cell types. The epithelial cluster was subsetted for further analysis of epithelial subtypes using established markers (Cooley et al., 2023). Differential gene expression was assessed between OVX Control and OVX P4 samples by downsampling to the smallest group size and applying the Seurat ‘FindMarkers’ function. To evaluate TF activity at single-cell resolution, the SCENIC workflow (Aibar et al., 2017) was applied and differential activity was quantified using AUCell scores analyzed with the limma package (Ritchie et al., 2015).

### Quantification and statistical analysis

Statistical analyses were performed using standard parametric and non-parametric tests as appropriate for each dataset. For murine inflammatory gene expression studies, relative mRNA levels of *Tnf*, *Il6*, *Il1b* and *Cxcl2* in uterine and cervical tissues from WT and Olfm4 knockout mice exposed to intraperitoneal LPS (5 mg/kg, 6 h) versus PBS control (*n*=5 per condition) were compared using unpaired two-tailed *t*-tests, with a significance threshold of *P*<0.05. PTB outcomes following vaginal inoculation with live pathogenic *E. coli* (10^7^ CFU) and intraperitoneal LPS administration (5 mg/kg) were compared between WT and knockout mice using Fisher's exact test. *E. coli*-induced PTB in WT (*P*=0.20) and knockout mice (*P*=0.94) were compared using Fisher's exact test. Sample size was determined based on the standard practice of murine models of LPS-induced inflammation and *E. coli* induced PTB, where group sizes of *n*=5 have shown to provide sufficient power to detect significant differences in gene expression and pregnancy outcomes (Akgul et al., 2014).

## Supplementary Material



10.1242/develop.205710_sup1 Supplementary information
