# Supplementary material for: Olfactomedin 4 marks progenitor cells that give rise to the secretory cell lineage in the mouse cervix
Source: Development. 2026 Oct 12;153(19):dev205710. doi: 10.1242/dev.205710 (PMC13723408; doi:10.1242/dev.205710)
Supplement: Supplementary file 1 [file develop-153-205710-s1.pdf]

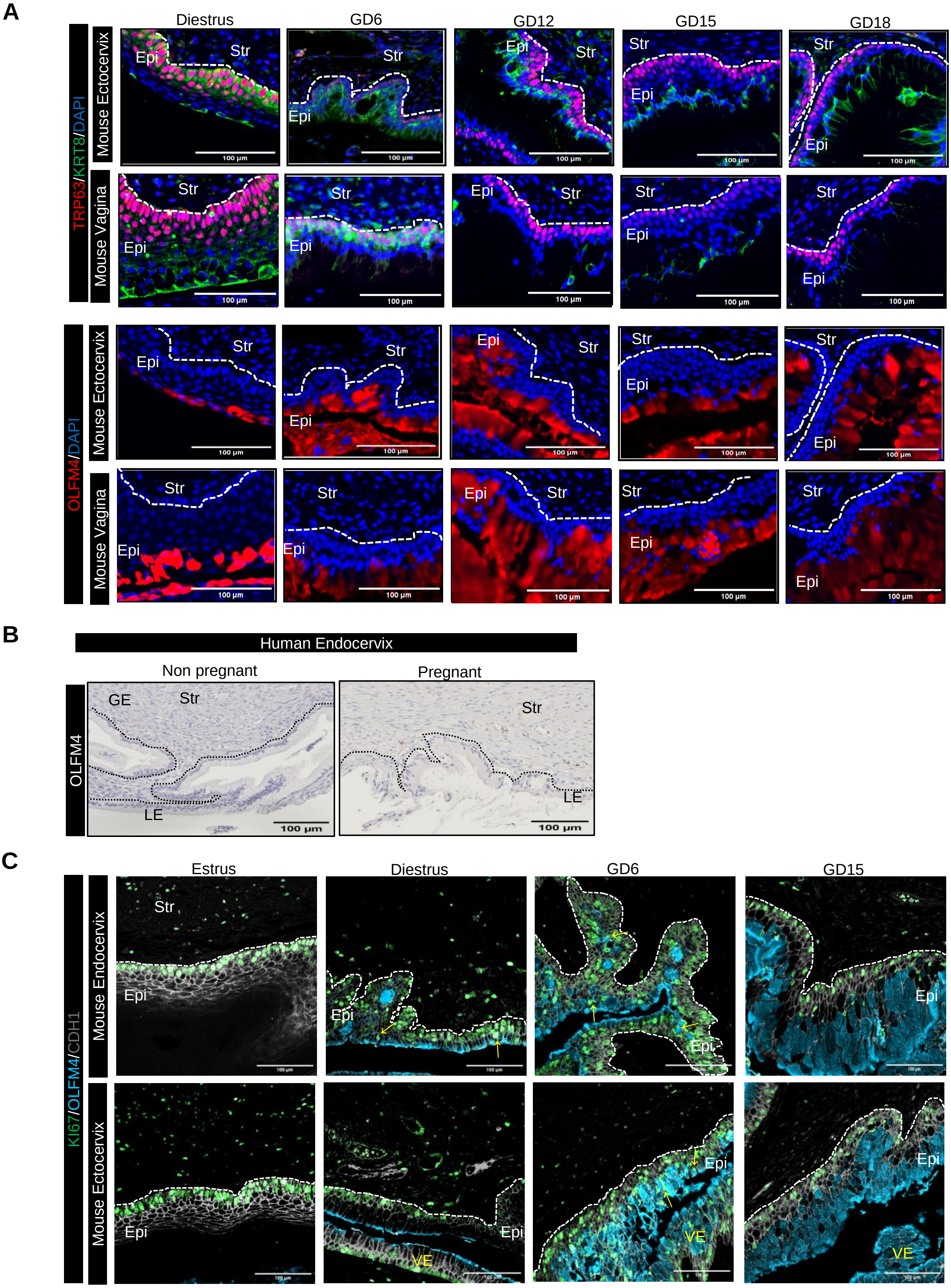
A
Diestrus
GD6
GD12
GD15
GD18
TRP63/KRT8/DAPI
Mouse Ectocervix
Mouse Vagina
OLFM4/DAPI
Epi
Str
100 µm
B
Human Endocervix
Non pregnant
Pregnant
OLFM4
GE
LE
C
Estrus
Diestrus
GD6
GD15
KI67/OLFM4/CDH1
Mouse Endocervix
Mouse Ectocervix
VE

Fig. S1. Distinct epithelial subtypes express squamous (TRP63) or columnar (KRT8/OLFM4) markers in the ectocervix and vagina of NP and pregnant mice.

A. Immunofluorescence staining on transverse sections of the ectocervix and vagina across non-pregnant (diestrus), pregnant (GD6, GD12, GD15, GD18) timepoints. Top panels show TRP63 (red), KRT8 (green), and nuclei (blue); bottom panels show OLFM4 (red) and nuclei (blue), illustrating temporal and spatial expression across the same timepoints. Images are representative of n = 3 biological replicates per time point, and for each marker combination the endocervix (Figure 1), ectocervix and vagina images in Figure S1 are taken from the same tissue section. Scale bars = 100 µm.

B. Immunohistochemical staining for OLFM4 in human endocervix. N= 2 biological replicates per group. Scale bar 100 µm. LE: luminal epithelia, GE: glandular epithelia, Str:Stroma.

C. Immunofluorescence staining for KI67 (green), OLFM4 (cyan), and CHD1 (grey) in the mouse cervix at estrus, diestrus, GD6, and GD15 timepoints. Images show endocervix (upper rows) and ectocervix (lower rows) with endo and ectocervical images taken from the same tissue section. Epi-epithelial, Str- stromal regions, VE-Vaginal epithelia and colocalization of KI67 and Olfm4 (yellow arrow) are indicated. n=3 biological replicates per time point and scale bars: 100 µm, Magnification: 40X.

Development • Supplementary information

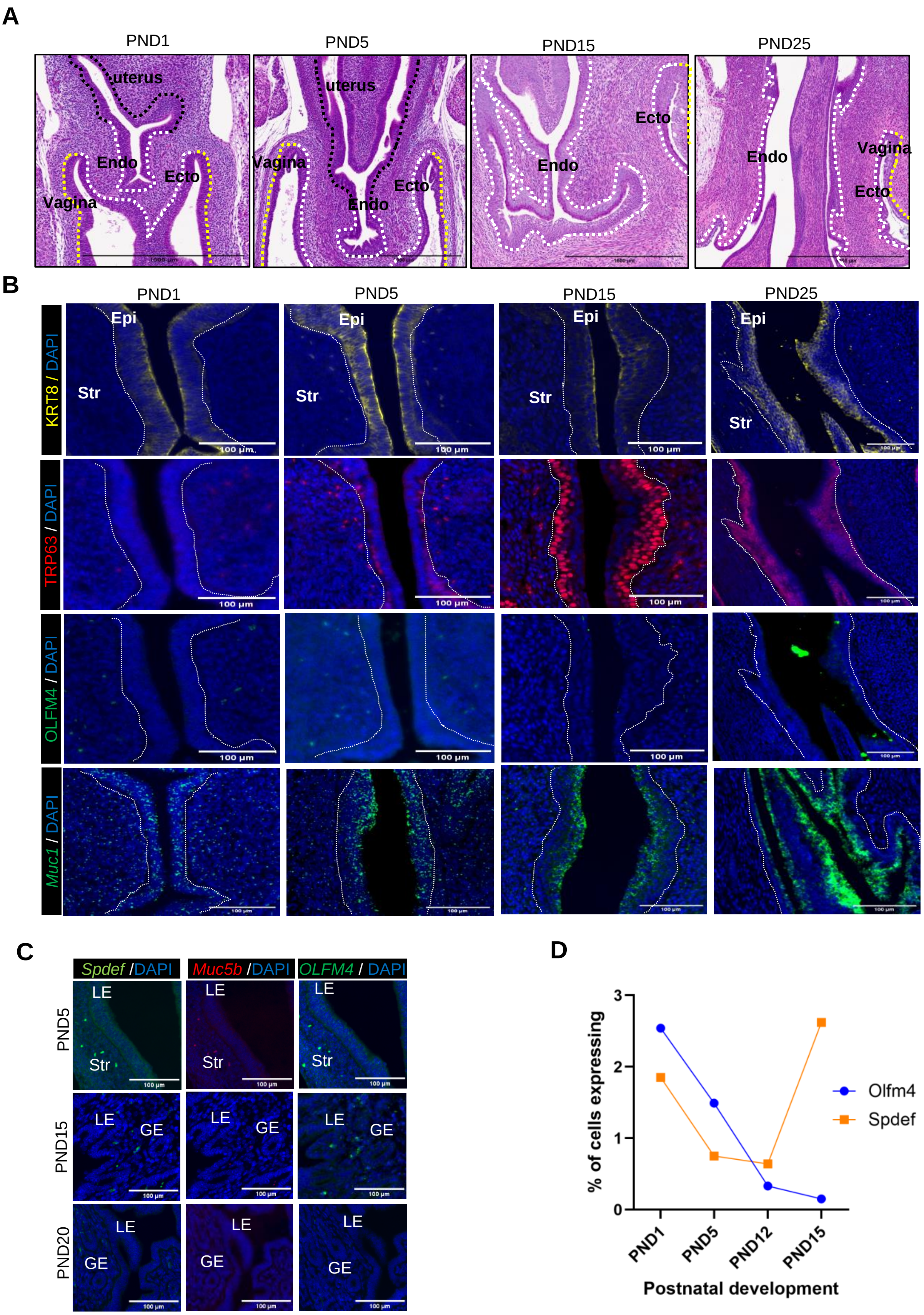

Development • Supplementary information

Fig. S2. H&E and RNA expression in the mouse female reproductive tract during development.

A. Hematoxylin and eosin (H&E) staining of FRT at postnatal day (PND) 1, 5, 15, and 25. Anatomical compartments are outlined with colored dotted lines: cervix (white), and vagina (yellow). Scale bars = 1000 µm. Ecto, ectocervix; Endo, endocervix.

B. Magnified images taken from images in Figure 2A.

C. Spdef, Muc5b and Olfm4 are not expressed in the uterus during the postnatal developmental period. Magnified images are taken from the same sections as shown in Figure 2B. LE: Luminal epithelia; GE: Glandular epithelia. scale bars: 100 µm.

D. Line graph showing the percentage of cells expressing Olfm4 and Spdef in PND 1,5,12,15 uterine tissue as described in (Spencer et al., 2023).

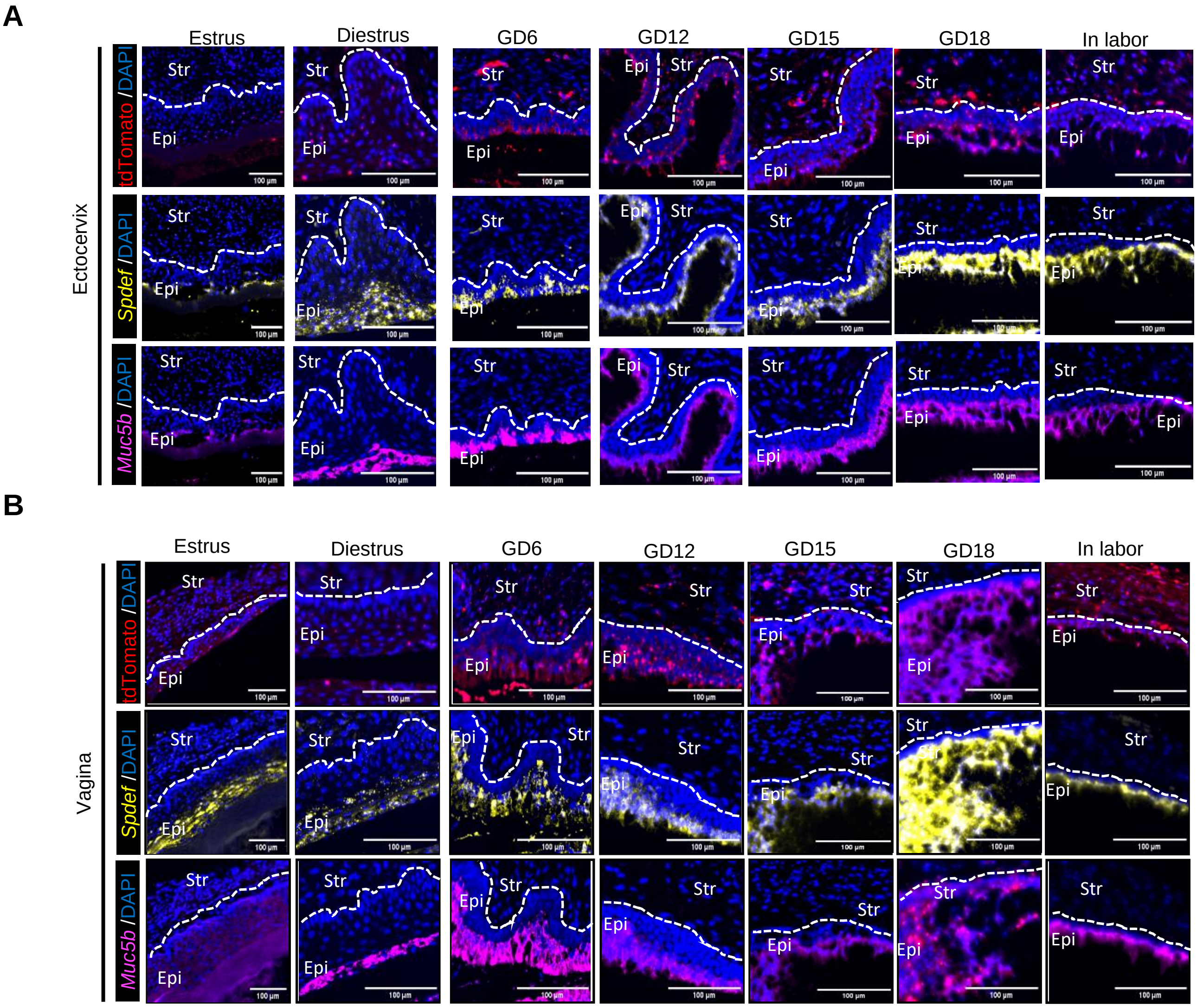

Fig. S3. Krt8⁺ progenitors give rise to goblet cells in the ectocervix and vagina.

A. RNAscope staining in the ectocervix for tdTomato (red, top panel), Spdef (yellow, middle panel), and Muc5b (magenta, bottom panel) across estrus, diestrus, pregnancy stages and in labor. Spdef and Muc5b were co-stained on the same tissue section and are shown in separate panels, whereas tdTomato was stained on a second section from the same animal. Images are representative of n=2 biological replicates per time points. Scale bars =100 µm.

B. RNAscope staining in the vagina for tdTomato (red, top panel), Spdef (yellow, middle panel), and Muc5b (magenta, bottom panel) across estrus, diestrus, pregnancy stages and in labor. Spdef and Muc5b were co-stained on the same tissue section and are shown in separate panels, whereas tdTomato was stained on separate section. Images are representative of n=2 biological replicates per time points. Scale bars =100 µm.

Development • Supplementary information

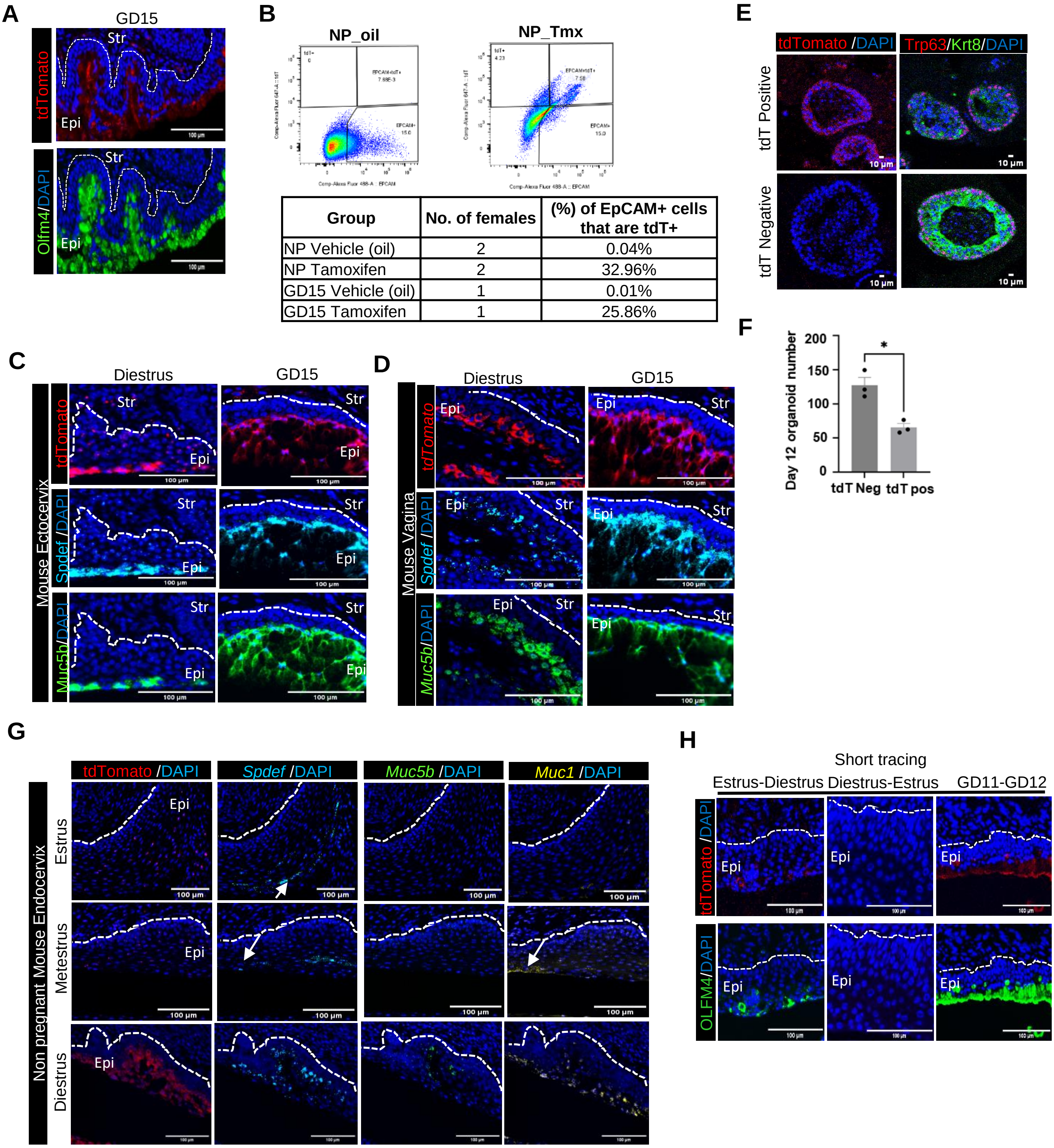

Group	No. of females	(%) of EpCAM+ cells that are tdT+
NP Vehicle (oil)	2	0.04%
NP Tamoxifen	2	32.96%
GD15 Vehicle (oil)	1	0.01%
GD15 Tamoxifen	1	25.86%

Fig. S4. Olfm4+ progenitors give rise to goblet cells in cervix and vagina in nonpregnant and pregnant states.

A. Co-stain for tdTomato and Olfm4 protein on the same gestation D15 cervix section. Image is shown in two separate panels. N=2 biological replicates. Scale bar = 100um.

B. Representative flow cytometry plots of dissociated cervix from Olfm4-CreERT2; Rosa26-tdTomato females treated with vehicle (corn oil; NP_oil) or tamoxifen (NP_Tmx), showing EpCAM (Alexa Fluor 488, x-axis) versus tdTomato (Alexa Fluor 647, y-axis) after gating on live single cells. Quadrants indicate $EpCAM^+$ (epithelial), $EpCAM^+tdT^+$ (labeled epithelial), and tdT^+ (non-epithelial) populations; values are percent of live cells. The table summarizes the proportion of epithelial cells carrying the reporter, calculated as tdT^+EpCAM^+ divided by total $EpCAM^+ \times 100$ (mean; n = number of females indicated). Tamoxifen induced tdTomato in 32.96% of $EpCAM^+$ cells in non-pregnant and 25.86% in pregnant (GD15) females, whereas vehicle controls showed ≤0.04% in both states, confirming that reporter activation is tamoxifen-dependent with minimal background labeling. Non-pregnant groups, n = 2; pregnant groups, n = 1.

C. RNA expression of tdTomato+ (red), Spdef (cyan) and Muc5b (green) in ectocervical tissue from nonpregnant (diestrus) and GD15. All three markers were detected in the same section, with each signal displayed in separate panels. Images are representative of n≥ 3 biological replicates per time point and scale bars =100 µm.

D. RNA expression of tdTomato+ (red), Spdef (cyan) and Muc5b (green) in vaginal tissue from nonpregnant (diestrus) and GD15. All three markers were detected in the same section, with each signal displayed in separate panels. Images are representative of n≥ 3 biological replicates per time point and scale bars =100 µm.

E. Immunofluorescent staining of tdT positive and tdT negative organoids. Left panels show tdTomato (red) with nuclear counterstain DAPI (blue). Right panels show co-staining for Trp63 (red) and Krt8 (green) with DAPI (blue). Scale bars: 10 µm.

F. Bar graph shows the mean number of organoids formed on Day 12 from tdTomato negative and tdTomato positive cells. Individual points represent n = 3 independent experiments per group. Statistical analysis was performed using a Welch's t-test, with $p<0.05$ considered significant

G. RNAscope staining of lineage-traced $tdTomato^+$ (red) cells with goblet cell markers Spdef (cyan), Muc5b (green), and Muc1 (yellow) across three stages of the nonpregnant endocervix estrus cycle. Representative of n = 3 biological replicates per time point. Scale bars = 100 µm.

H. Co-stain for tdTomato and OLFM4 protein on the same section in the short-tracing study. Image is shown in two separate panels. N=2 biological replicates. Scale bar = 100um.

Development • Supplementary information

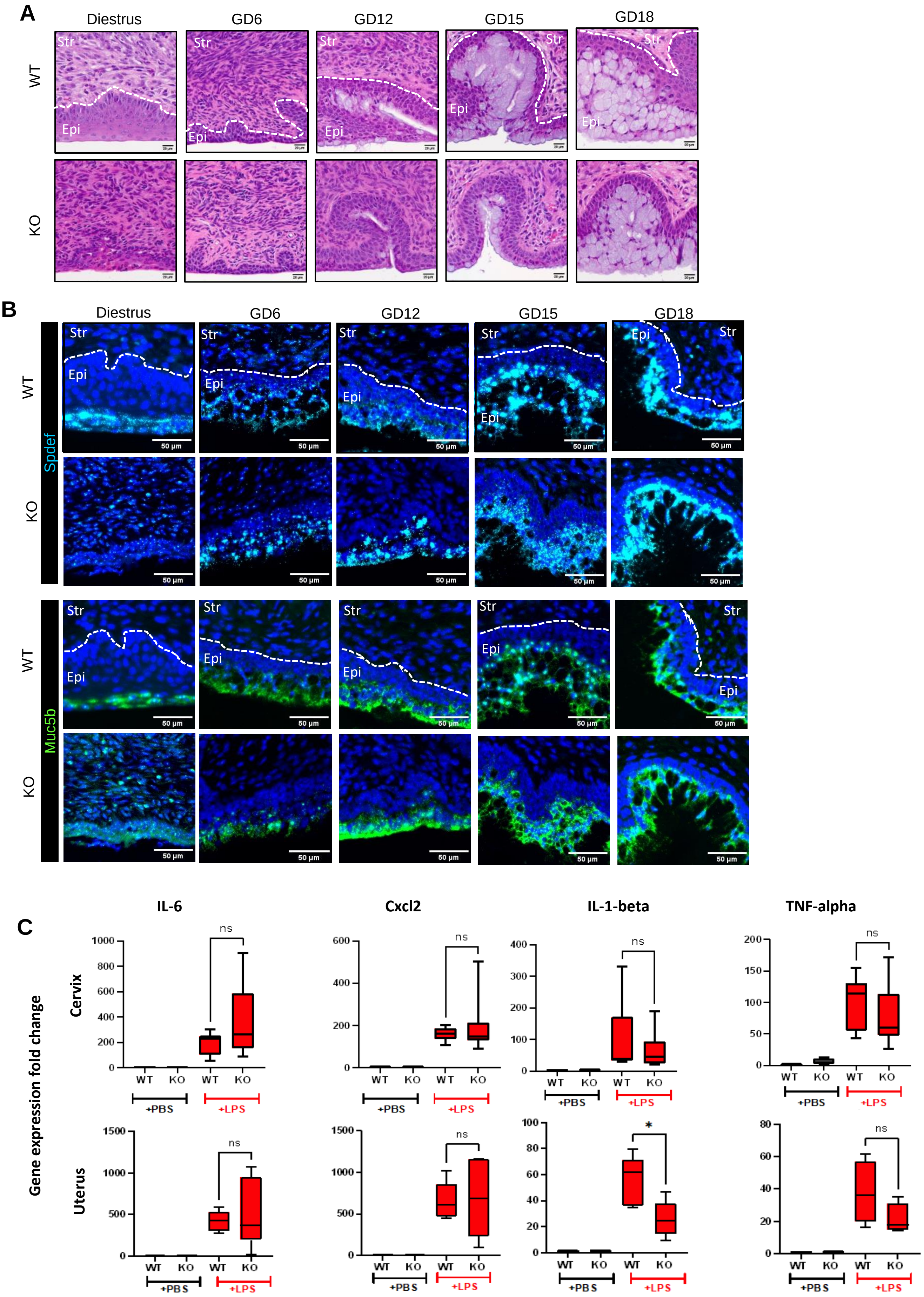

Development • Supplementary information

Fig. S5. Deficiency in Olfm4 does not affect cervical goblet morphology and function

A. Hematoxylin and eosin (H&E) staining of the endocervical canal in Olfm4 wild-type (WT) and knockout (KO) mice during diestrus and at gestational day 6, 12, 15, 18 timepoints. Representative images from n = 2 biological replicates per genotype and timepoint. Scale bar =20 µm.

B. RNAscope staining for transcription factor Spdef (cyan), Muc5b (green), nuclei (blue) in WT and KO endocervical tissues at nonpregnant diestrus and gestational day 6, 12, 15, 18 timepoints. A single section was stained for the 3 markers and each marker displayed separately in the panel. Representative images from n = 1 biological replicates per condition. Scale bar =50 µm.

C. Gene expression of IL6, Cxcl2, IL1-beta, TNF-alpha inflammatory markers in the cervical (top panel) and uterine tissue (bottom panel) of WT and Olfm4 KO mice exposed to intraperitoneal injection (IP) of lipopolysaccharide (LPS, 5 mg/kg) as compared to IP PBS for 6 hours. (n=5 per condition). Comparisons were performed using the unpaired *t*-test analysis, with $p<0.05$ considered significant.

Development • Supplementary information

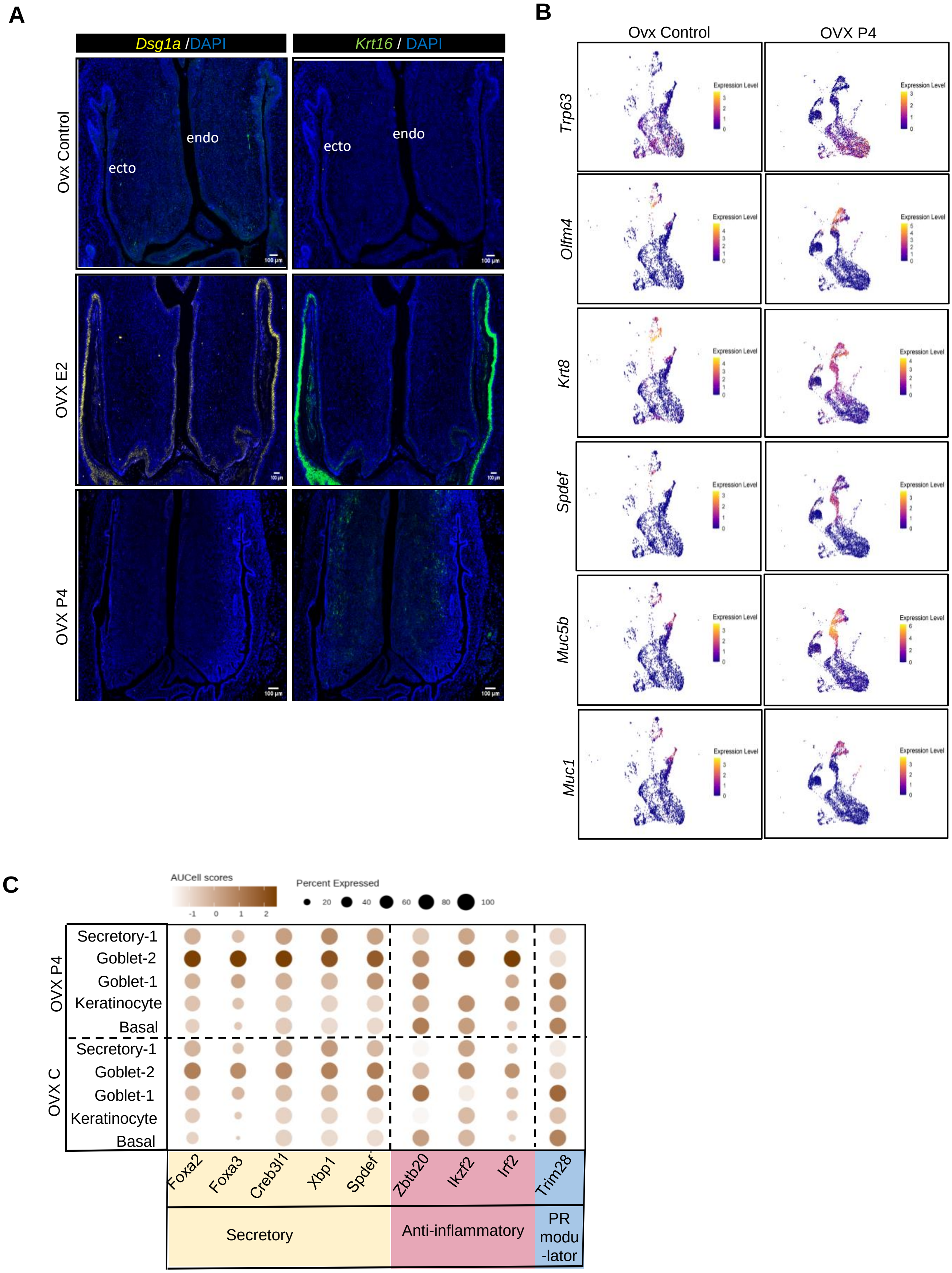
A
Dsg1a /DAPI
Krt16 / DAPI
Ovx Control
OVX E2
OVX P4
endo
ecto
100 µm
B
Ovx Control
OVX P4
Trp63
Olfm4
Krt8
Spdef
Muc5b
Muc1
Expression Level
C
AUCell scores
-1 0 1 2
Percent Expressed
20 40 60 80 100
OVX P4
OVX C
Secretory-1
Goblet-2
Goblet-1
Keratinocyte
Basal
Foxa2
Foxa3
Creb3l1
Xbp1
Spdef
Zbtb20
Ikzf2
Irf2
Trim28
Secretory
Anti-inflammatory
PR modu -lator

Fig. S6. Progesterone induces Olfm4+ progenitor differentiation into goblet cells

A. RNAscope staining of the lower female reproductive tract (cervix and vagina) for Dsg1a (yellow, left panels) and Krt16 (green, right panels), with nuclei (blue): OVX Control (no hormone), OVX E2 (estradiol), and OVX P4 (progesterone). Both markers were stained on the same sections and are shown in separate panels. Images are representative of n = 3 biological replicates per condition. Scale bars = 100 µm.

B. Feature plots from scRNA-seq showing expression of *Trp63*, *Olfm4*, *Krt8*, *Spdef,* and *Muc5b, Muc1* across epithelial clusters in OVX Control and OVX P4 cervices.

C. SCENIC analysis predicting transcription factor regulons activated in OVX P4 group epithelial clusters. Transcription factors are grouped by function: secretory associated (cream), anti-inflammatory (pink), modulators of progesterone receptor (PR) activity (blue). The matrix highlights cluster-specific regulon activity, including Foxa2/3, Creb3l1, and Spdef in secretory clusters, Zbtb20 and Irf2 in anti-inflammatory clusters, and Trim28 in PR-modulatory clusters.

Development • Supplementary information

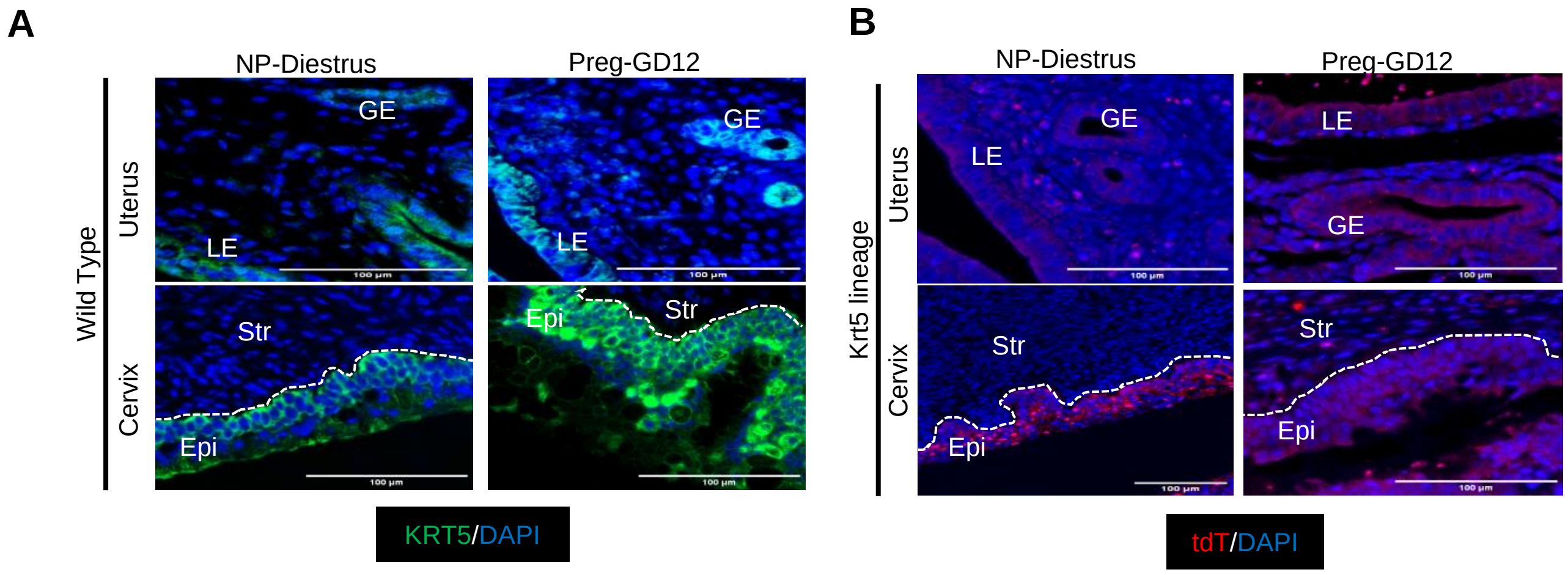

Fig. S7. Keratin 5 is not a faithful marker of the Trp63 basal progenitor lineage.

A. Immunofluorescence staining of KRT5 (green) in the uterus (top panels) and cervix (bottom panels) of wild-type mice at diestrus and gestational day (GD) 12, illustrating spatial KRT5 expression across the two tissues. Images are representative of n = 2 biological replicates per timepoint. LE: luminal epithelia; GE: glandular epithelia; Str: Stroma; Epi: epithelia.Scale bars, 100 µm.

B. Immunofluorescence staining of tdTomato (red) in the uterus (top panels) and cervix (bottom panels) of Krt5-CreERT2; Rosa26-tdTomato lineage-tracing mice at diestrus and gestational day (GD) 12, illustrating the spatial distribution of Krt5-lineage (tdTomato^{+}) cells across the two tissues. Images are representative of n = 2 biological replicates per timepoint. Scale bars, 100 µm.

Development • Supplementary information

Table S1. Preterm birth outcomes in WT and Olfm4 KO mice following E.coli inoculation. Related to Figure 5.

	E. coli (10^7 CFU)				IP LPS	
**Group**	**Term**	**Preterm**	**PTB rate**	**n**	**Mean time to delivery (hours)**	**n**
**WT**	10	10	50%	20	13	9
**KO**	13	5	28%	18	11	9

Table S2. The epithelial subtype proportions in OVX Control vs OVX P4 samples. Related to Figure 6.

Cell type	OVX control		OVX P4	
	**n cells**	**%**	**n cells**	**%**
**Basal**	1354	41.0	2408	37.5
**Keratinocytes**	737	22.3	1174	18.3
**Goblet-1**	133	4.0	1300	20.2
**Goblet-2**	298	9.0	1468	22.8
**Secretory-1**	781	23.6	79	1.2
**Total**	**3303**		**6429**	
