# Supplementary material for: Olfactomedin 4 marks progenitor cells that give rise to the secretory cell lineage in the mouse cervix
Source: Development. 2026 Oct 12;153(19):dev205710. doi: 10.1242/dev.205710 (PMC13723408; doi:10.1242/dev.205710)
Supplement: Supplementary file 2 [file develop-153-205710_review_history.pdf]

Olfactomedin 4 marks progenitor cells that give rise to the secretory cell lineage in the mouse cervix

ShanmugaPriyaa Madhukaran, Yevgenia Fomina, Govindkumar Balagannavar, Elizabeth Payne, Jason Wilson, Lei Wang, Gary Hon, Maria Florian Rodriguez and Mala Mahendroo

DOI: 10.1242/dev.205710

Editor: Liz Robertson

Review timeline

Original submission:	31 March 2026
Editorial decision:	27 May 2026
First revision received:	22 July 2026
Editorial decision:	11 August 2026
Second revision received:	27 August 2026
Accepted:	3 September 2026

Original submission

First decision letter

MS ID#: dev.205710

MS TITLE: Olfactomedin 4 marks luminal progenitor cells that give rise to a secretory cell lineage in the mouse cervix

AUTHORS: ShanmugaPriyaa Madhukaran; Yevgenia Fomina; Govindkumar Balagannavar; Elizabeth Payne; Jason Wilson; Lei Wang; Gary Hon; Maria Florian Rodriguez; Mala Mahendroo

Dear Dr Mahendroo,

I have now received all the referees' reports on the above manuscript, and have reached a decision. The referees' comments are appended below.

As you will see, the referees express considerable interest in your work, but Reviewer 1 has some significant criticisms and recommend revision of your manuscript before we can consider publication. If you are able to revise the manuscript along the lines suggested, which may involve further experiments, I will be happy receive a revised version of the manuscript. Your revised paper will be re-reviewed by one or more of the original referees, and acceptance of your manuscript will depend on your addressing satisfactorily the reviewers' major concerns. Please also note that Development will normally permit only one round of major revision. If it would be helpful, you are welcome to contact us to discuss your revision in greater detail. Please send us a point-by-point response indicating your plans for addressing the referees' comments, and we will look over this and provide further guidance.

Please attend to all of the reviewers' comments and ensure that you upload both a 'clean' version of your Word file, along with a highlighted version clearly showing where you have made changes in the revised manuscript. Please avoid using 'Tracked changes' in Word files as these are lost in PDF conversion. I should be grateful if you would also provide a point-by-point response detailing how

© 2026. Published by The Company of Biologists under the terms of the Creative Commons Attribution License (https://creativecommons.org/licenses/by/4.0/).

you have dealt with the points raised by the reviewers in the 'Response to Reviewers' box. If you do not agree with any of their criticisms or suggestions please explain clearly why this is so.

Reviewer 1

SUMMARY OF THE ADVANCE MADE IN THIS PAPER AND ITS POTENTIAL SIGNIFICANCE TO THE FIELD

This manuscript seeks to understand the cellular origins of mucus-producing (goblet) cells in the mouse cervix. This is a critical question due to the role of these cells in producing antimicrobials that guard against infections that can cause preterm birth. Very little work on the lineage relationships in the cervix has been done, and this study takes an important step towards filling this gap.

The authors identify Olfm4 as a marker of a subset of Krt8+ cells in the adult cervix (in mice - they find no Olfm4 in human cervix). They characterize the emergence of these Olfm4+ cells during postnatal development and find that Olfm4+ cells arise between PND5 and PND15. By inducing recombination with an Olfm4-CreER in non-pregnant animals and then getting them pregnant, they find that the labeled population expands during both a first and second pregnancy, suggesting long-term progenitor potential of this population. (Both Olfm4+ and - cells also have organoid-forming capacity). They also show that Olfm4+ cells expand in response to P4 administration to ovx mice. Importantly, they also show that Olfm4 itself is not important for goblet cell function.

The key model proposed here is that there are 2 progenitor cells in the adult mouse cervix: a squamous TP63+ cell that makes basal cells and an Olfm4+ cell that makes goblet cells. The data for this are relatively compelling, particularly the 2-pregnancy lineage tracing. There are some experiments that I believe would provide stronger support for the 2-progenitor model, and if they are addressed I am supportive of publication in Development.

SUGGESTIONS TO AUTHORS

Major concerns and suggestions:

1. Olfm4-CreER; tdTomato lineage tracing is used to show progenitor potential of this cell population. A critical consideration is how leaky the Cre and the reporter are - if non-Olfm4 cells are spuriously turning on TdTomato, this makes it very challenging to interpret red cells as being derived from Olfm4+ cells.

a. An important control for this is that the proportion of red epithelial cells in Olfm4-CreER; tdTomato animals given corn oil instead of tamoxifen must be quantified (particularly given corn oil and then progressed through pregnancy) to understand what the background labeling is. (This could be efficiently quantified by FACS, or less efficiently by manual counting from microscopy)/

b. Further, although they say "the spatial distribution of tdTomato+ expression in the cervix closely mirrored endogenous OLFM4 expression… confirming reporter fidelity", this inference is indirect and co-staining of TdTomato and Olfm4 at early time points should be performed to determine what proportion of TdTomato+ cells are Olfm4+.

c. Most compelling would be to do a "negative" lineage tracing experiment, i.e. to show that Olfm4 cells are not derived from TP63 cells. To my knowledge, there is no appropriate Tp63-Cre available at Jax, and I do not think it is essential to acquire it. However, my understanding from the Fig. 1 cartoon and from PMID 34719766 is that Krt5 also marks the TP63 cell population, and a Krt5-CreER is available live at Jax. The ideal experiment would be to show that goblet cells are not derived from a K5 progenitor by lineage tracing with this mouse. However, as this requires additional genetic crossing, I leave it to the editors to decide if this is necessary. I note that this strain is widely used by skin labs who may already have the correct genotype and may be able to send you the cervix of pregnant mice to save you the crosses.

© 2026. Published by The Company of Biologists under the terms of the Creative Commons Attribution License (https://creativecommons.org/licenses/by/4.0/).

i. In PMID 34719766, they show that MUC1-positive cells are Krt5-Cre traced, although I find this a bit hard to interpret from their images. Nonetheless, this seems to be in conflict with the model proposed here that MUC1 cells are Olfm4 derived (Fig S4).

d. In a truly ideal world, depletion of the Olfm4-positive cells would also be performed (i.e. Olfm4-CreER; DTA or DTR). One would expect this to abrogate goblet cell production. The DTA/DTR mice are also available live at Jax, and again I leave it to the editors to assess the necessity of these experiments.

2. Broadly: Terms such as "simple columnar," "stratified squamous," "secretory," "goblet," "secretory luminal," "columnar," "basal progenitors," and "secretory epithelium" are used inconsistently and sometimes interchangeably. This makes it difficult to follow the relationships between cell types across figures and datasets. I recommend standardizing the terminology throughout the text and figures, and explicitly defining each epithelial subtype (e.g., in a schematic or table) with both histologic and molecular criteria.

a. More specifically, I am overall confused about the relationship of the non-goblet secretory/MUC1 cells to the Olfm4 cells. Based on Figure 1B, it looks as though, in the non-pregnant sample, it is not goblet cells that are Olfm4 positive, but rather the secretory IL and secretory NP cells. Presumably, these are the cells being traced in subsequent lineage tracing experiments. This should be clarified. Are all secretory (non-goblet) cell populations in the single cell data (Fig 1) MUC1 positive i.e. are these cells Olfm4+Muc1+? If so, the data in Fig. S4D that cells are tdTom+Muc1+ would be because the cells are double positive, not necessarily because they went through an Olfm4 -> Muc1 transition. Things that would help clarify the Olfm4/Muc1/other secretory relationship:

i. Fig 1B and Fig 6c need feature plots for MUC1.

ii. Clear discussion of how Olfm4- secretory cells fit into the proposed model (and whether these are all MUC1+)

1. Figure 2a shows quite a lot of Krt8+ Olfm4- cells - are these the Muc1+ cells? An intermediate?

iii. Revise language to make it clear that Olfm4 is not strictly goblet-specific.

Minor comments:

1. Terminology and annotation

i. Looking at Figure 1B, there appears to be large goblet populations (goblet NPIL and goblet 2) that are Olfm4 negative and do not seem to be referenced.

ii. The text mentions subclassification of luminal cells from the scRNAseq data into goblet, Muc1 secretory cells and keratinocyte-like cells, but this is not clearly represented in the UMAP in Fig 1B. Are the cells labeled as luminal cells in the UMAP the keratinocyte-like cells?

iii. Figure 1B and 6C need explanation of the annotation for both figures (with feature plots), and the correspondence of clusters (or not) between the Fig 1B and 6C should be discussed.

2. The setup of the experiment showing persistence and expansion of tdTomato cells after ovx and P4 treatment (Fig S4E) is not clear to me and is in need of a schematic. When were the animals given tamoxifen relative to the ovx, what was the E2 and P4 regimen, and how long after ovx were they characterized?

a. As an aside, this experiment seems highly related to the experiments in figure 6 and perhaps would be best combined with that figure and expanded, as figure 6 claims to characterize "the impact of progesterone on the proliferation and differentiation of the olfm4+ luminal progenitor", but does not perform the relevant lineage tracing to show that this is expansion from the Olfm4+ progenitor rather than conversion of Olfm4- cells into a goblet fate.

© 2026. Published by The Company of Biologists under the terms of the Creative Commons Attribution License (https://creativecommons.org/licenses/by/4.0/).

3. It is very hard to assess proliferation of Olfm4 positive cells from the data provided in S1C, in part because the Olfm4 staining is so strong on the apical surface and far away from the nuclei.

4. Figure 1a: presumably the dots in the figure are glands - please describe in the legend or remove.

5. Figure 4a: please update to clarify how many days after tamoxifen injection the diestrus (NP) sample was harvested.

6. Figure 5A, GD18 WT is spectacular and should be entered into a microscopy competition and/or framed on a wall.

Reviewer 2

SUMMARY OF THE ADVANCE MADE IN THIS PAPER AND ITS POTENTIAL SIGNIFICANCE TO THE FIELD

The manuscript by Madhukaran and coworkers investigates the cell lineage of the mouse cervix. Current dogma states that while the human cervix has two progenitor cells, the columnar and basal cell the mouse cervix has one progenitor cell the basal TP63+ cell. This manuscript shows by immunohistochemistry, single Cell RNA Seq analysis and cell lineage tracing that the mouse has two progenitor cells, The Tp63+ cell and the Krt8+ cell They also demonstrate that the Olfactomedian 4+ , OLFM4+, is a marker for the Krt8+ cell population . They demonstrate that the OLMF4+ cell population is initiated during cervical postnatal development, is increased during pregnancy , induced by progesterone and is a self-renewing population. They also demonstrate that OLMF4 is not required for normal cervical function but serves as a marker for a specific population of cells in the cervix. This report does acknowledge that although it proposes to progenitor cell populations the Tp63+ and The Krt8+ population it does not rule out a Double positive progenitor cell and does acknowledge that lineage tracing with a TP63Cre model is the next step.

All in all this is a well designed and executed set of experiment that sets a new paradigm for cervical development in the mouse. It demonstrates that the mouse cervix cell populations align more with the human cervix and presents an in vivo model to investigate the changes in the cervix during pregnancy.

First revision

Author response to reviewers' comments

We thank the reviewers for the careful evaluation of our work and are extremely appreciative of the constructive comments and suggestions to improve the rigor and clarity of our work. We believe the manuscript is significantly improved based on the constructive feedback we received. Below we provide point by point responses to summarize how we addressed each comment.

Reviewer 1: SUMMARY OF THE ADVANCE MADE IN THIS PAPER AND ITS POTENTIAL SIGNIFICANCE TO THE FIELD.

This manuscript seeks to understand the cellular origins of mucus-producing (goblet) cells in the mouse cervix. This is a critical question due to the role of these cells in producing antimicrobials that guard against infections that can cause preterm birth. Very little work on the lineage relationships in the cervix has been done, and this study takes an important step towards filling this gap. The authors identify Olfm4 as a marker of a subset of Krt8+ cells in the adult cervix (in mice - they find no Olfm4 in human cervix). They characterize the emergence of these Olfm4+ cells during postnatal development and find that Olfm4+ cells arise between PND5 and PND15. By inducing recombination with an Olfm4-CreER in non-pregnant animals and then getting them

© 2026. Published by The Company of Biologists under the terms of the Creative Commons Attribution License (https://creativecommons.org/licenses/by/4.0/).

pregnant, they find that the labeled population expands during both a first and second pregnancy, suggesting long-term progenitor potential of this population. (Both Olfm4+ and - cells also have organoid-forming capacity). They also show that Olfm4+ cells expand in response to P4 administration to ovx mice. Importantly, they also show that Olfm4 itself is not important for goblet cell function. The key model proposed here is that there are 2 progenitor cells in the adult mouse cervix: a squamous TP63+ cell that makes basal cells and an Olfm4+ cell that makes goblet cells. The data for this are relatively compelling, particularly the 2-pregnancy lineage tracing. There are some experiments that I believe would provide stronger support for the 2-progenitor model, and if they are addressed, I am supportive of publication in Development.

SUGGESTIONS TO AUTHORS

Major concerns and suggestions.

1. Olfm4-CreER; tdTomato lineage tracing is used to show progenitor potential of this cell population. A critical consideration is how leaky the Cre and the reporter are - if non-Olfm4 cells are spuriously turning on TdTomato, this makes it very challenging to interpret red cells as being derived from Olfm4+ cells.

a. An important control for this is that the proportion of red epithelial cells in Olfm4-CreER; tdTomato animals given corn oil instead of tamoxifen must be quantified (particularly given corn oil and then progressed through pregnancy) to understand what the background labeling is. (This could be efficiently quantified by FACS, or less efficiently by manual counting from microscopy).

We thank the reviewer for this important point. We agree that distinguishing tamoxifen-induced recombination from tamoxifen-independent background is an important control.

Olfm4-CreERT2; Rosa26tdTomato females received either corn oil vehicle or tamoxifen [induction regimen, Fig. 4A], and cervical epithelial cells were quantified by flow cytometry using antibody-based detection of tdTomato and EpCAM as described in the method section. Within the EpCAM$^+$ live epithelial compartment, tdTomato$^+$ cells comprised 0.01-0.04% in vehicle-treated animals compared with 33% in tamoxifen-treated nonpregnant mice and 26% of epithelia were tdT+ in tamoxifen-treated GD15 pregnant mice. The near-absence of labeling in the vehicle condition indicates that recombination in the cervical epithelium is tamoxifen-dependent, with minimal intrinsic leakiness of the Cre or reporter allele.

These data have been added as a new panel in Supplemental Figure S4B (FACS gating strategy and quantification) and are referenced in the Results section describing the Olfm4 lineage tracing. The Methods section has been updated to include the vehicle control experimental design.

b. Further, although they say "the spatial distribution of tdTomato+ expression in the cervix closely mirrored endogenous OLFM4 expression… confirming reporter fidelity", this inference is indirect and co-staining of TdTomato and Olfm4 at early time points should be performed to determine what proportion of TdTomato+ cells are Olfm4+.

We agree that direct co-staining of tdTomato and endogenous OLFM4 provides a more rigorous demonstration of reporter fidelity than the indirect spatial comparison originally presented.

We performed dual immunofluorescence for tdTomato and OLFM4 on sections from timepoints in pregnancy in which tamoxifen was administered prior to pregnancy (Fig 4A) and our short- tracing experiments (Figure 4E), in which tamoxifen was administered and tissues harvested 24 to 48 hours later. As shown in new Supplemental Figures S4A and S4H, tdTomato$^+$ epithelial cells co-express endogenous OLFM4 protein at both long and short (24-48h) post-tamoxifen interval, directly supporting reporter fidelity at the Olfm4.

c. Most compelling would be to do a "negative" lineage tracing experiment, i.e. to show that Olfm4 cells are not derived from TP63 cells. To my knowledge, there is no appropriate Tp63-Cre available at Jax, and I do not think it is essential to acquire it. However, my understanding from the Fig. 1 cartoon and from PMID 34719766 is that Krt5 also marks the TP63 cell population, and a Krt5-CreER is available live at Jax. The ideal experiment would be to show that goblet cells are not

© 2026. Published by The Company of Biologists under the terms of the Creative Commons Attribution License (https://creativecommons.org/licenses/by/4.0/).

derived from a K5 progenitor by lineage tracing with this mouse. However, as this requires additional genetic crossing, I leave it to the editors to decide if this is necessary. I note that this strain is widely used by skin labs who may already have the correct genotype and may be able to send you the cervix of pregnant mice to save you the crosses.

We agree with the reviewer that the “negative” lineage experiment would be a valuable approach to support the inability of basal progenitors to give rise to goblet cells which ideally would be done using a Trp63 reporter. Since Trp63 Cre reporters are not commercially available, we had previously conducted lineage studies using a Krt5Cre reporter since prior literature suggested it to overlap with Trp63.

To our surprise, lineage tracing results, mRNA expression and Krt5 protein staining by IF collectively suggested Krt5 is NOT a specific marker for cells derived from a basal progenitor lineage. Using our wild type mouse line, IF staining for Krt5 identified low Krt5 expression in the uterine luminal and glandular epithelium (Supp Fig 7A) in contrast to high expression in the corresponding cervix. We initially thought the uterine staining was background but subsequent lineage studies using the Krt5 led to our revised interpretation. Specifically, lineage tracing using the Krt5 reporter identified TdTomato+ cells in the uterine and cervical epithelia (Supp Fig 7B). Collectively this suggests that Krt5 is not a specific marker of the basal lineage in the female reproductive tract. Consistent with our data, KRT5 expression has also been recently described in the human endometrium, where KRT5+ cells form a distinct non-basal epithelial population in scRNA-seq analyses (Nikolakopoulou et al., Cell Stem Cell 2026).

We have included the described Krt5 studies in the revised manuscript in the discussion section and in (Supp Fig 7) as it may be of value to the field. We mention in the discussion the need to conduct future experiments using a Trp63 reporter and state that Krt5 which is considered to mark basal cells in the skin was found not to be specific to basal cells in the female reproductive tract. In addition, we have revised our model in Figure 1A by changing “Trp63, Krt5” as a marker of the basal progenitors to “ Trp63” only.

i. In PMID 34719766, they show that MUC1-positive cells are Krt5-Cre traced, although I find this a bit hard to interpret from their images. Nonetheless, this seems to be in conflict with the model proposed here that MUC1 cells are Olfm4 derived (Fig S4).

We suggest the findings in PMID 34719766 result from the broader expression of Krt5 in the female reproductive tract epithelium as described in our data above.

d. In a truly ideal world, depletion of the Olfm4-positive cells would also be performed (i.e. Olfm4-CreER; DTA or DTR). One would expect this to abrogate goblet cell production. The DTA/DTR mice are also available live at Jax, and again I leave it to the editors to assess the necessity of these experiments.

We agree that genetic depletion of Olfm4-positive cells using Olfm4-CreERT2; DTA or DTR crosses would provide direct functional evidence that the Olfm4+ progenitor is required for goblet cell production. However, generating these crosses and performing experiments across non- pregnant and pregnant time points represents a substantial undertaking beyond the scope of the current revision.

2. Broadly: Terms such as

"simple columnar,"

"stratified squamous," "secretory," "goblet," "secretory luminal," "columnar," "basal progenitors," and "secretory epithelium" are used inconsistently and sometimes interchangeably. This makes it difficult to follow the relationships between cell types across figures and datasets. I recommend standardizing the terminology throughout the text and figures, and explicitly defining each epithelial subtype (e.g., in a schematic or table) with both histologic and molecular criteria.

We appreciate the reviewer’s point and have sought to standardize terminology through the manuscript and provided a Table to define terminology.

© 2026. Published by The Company of Biologists under the terms of the Creative Commons Attribution License (https://creativecommons.org/licenses/by/4.0/).

a. More specifically, I am overall confused about the relationship of the non-goblet secretory/MUC1 cells to the Olfm4 cells. Based on Figure 1B, it looks as though, in the non-pregnant sample, it is not goblet cells that are Olfm4 positive, but rather the secretory IL and secretory NP cells. Presumably, these are the cells being traced in subsequent lineage tracing experiments. This should be clarified.

We apologize for creating this confusion. We have corrected the labeling of the Secretory IL to Goblet cell cluster in Fig 1B. The cluster we identified as "Secretory" in the NP did not express goblet cell markers but was Muc1+. Our subsequent lineage tracing studies at specific stages of the estrus cycle in the Olfm4 reporter (Supp Fig 4G) suggested that the cells labeled as Secretory may be an "early" cell state of goblet cell differentiation (more specifically these may be the Spdef+ Muc1+ cells seen in metestrus). We conclude that Olfm4 marks goblet cells and over the NP cycle the Olfm4+ cells undergo changes in cell states that are reflected by the distinct clusters in the scRNAseq data in Fig 1.

Are all secretory (non-goblet) cell populations in the single cell data (Fig 1) MUC1 positive i.e. are these cells Olfm4+Muc1+?

Yes, all the goblet cell/secretory cell clusters are Muc1+ though levels are very low in the "NP/IL Goblet" and "Goblet 2" clusters. As discussed in 2.a.ii. below, our current studies do not support the existence of an Olfm4 negative Goblet 2 cluster.

It is important to note that Muc1 is a marker for all secretory cells including the uterine luminal and glandular epithelia and is not unique to goblet cells.

If so, the data in Fig. S4D that cells are tdTom+Muc1+ would be because the cells are double positive, not necessarily because they went through an Olfm4 -> Muc1 transition. Things that would help clarify the Olfm4/Muc1/other secretory relationship:

i. Fig 1B and Fig 6c need feature plots for MUC1.

We now include Muc1 feature plots in Figure 1C and to Supplemental Figure S6B.

ii. Clear discussion of how Olfm4- secretory cells fit into the proposed model (and whether these are all MUC1+)

As presented in Figure 1C, all the goblet /secretory clusters express Olfm4 albeit at varying levels of expression. The cluster identified as "Goblet 2" in Fig 1B is the only Olfm4 negative cluster. Both our lineage studies and IF staining for Olfm4 failed to identify Olfm4 negative goblet cells, thus we do not have evidence that the Goblet 2 cluster in Fig 1B exists. We raise this point in the Discussion section and agree we needed to acknowledge this finding.

1. Figure 2a shows quite a lot of Krt8+ Olfm4- cells - are these the Muc1+ cells? An intermediate?

As suggested by the reviewer, we evaluated Muc1 expression in the postnatal period (Fig 2A). Based on this data it appears that the Krt8+Olfm4- cells are Muc1+. Thus the secretory cell marker, Muc1 is expressed at an earlier timepoint prior to the goblet specific marker, Olfm4.

iii. Revise language to make it clear that Olfm4 is not strictly goblet-specific.

Based on the requested clarifications to the manuscript and the addition of a schematic in Figure 7, we hope it is clear that we do consider Olfm4 to be a strict marker of goblet cells in the mouse cervix.

Minor comments:

1. Terminology and annotation

© 2026. Published by The Company of Biologists under the terms of the Creative Commons Attribution License (https://creativecommons.org/licenses/by/4.0/).

i. Looking at Figure 1B, there appears to be large goblet populations (goblet NPIL and goblet 2) that are Olfm4 negative and do not seem to be referenced.

Thank you for noting this omission. The NPIL cluster does have some Olfm4+ cells thus at present we consider them to be an "early" goblet cell state population. As discussed above, we provide some text in the Discussion to clarify that our prior annotation of the Olfm4- Goblet 2 cluster was based on expression of Krt8, Spdef and Muc5b. In the current studies we were unable to identify goblet cells that were not tdTomato+ or Olfm4+. Thus, our current data in this manuscript suggests that the cluster assigned as goblet 2 is unlikely to be goblet cells. We have included the following sentence in the Discussion: "The cluster assigned as "goblet 2" in Fig 1B are unlikely to be goblet cells and will require reassignment upon further investigation. Additionally, we have included a new schematic Figure 7 to clarify our revised model of two progenitors.

iii. The text mentions subclassification of luminal cells from the scRNAseq data into goblet, Muc1 secretory cells and keratinocyte-like cells, but this is not clearly represented in the UMAP in Fig 1B. Are the cells labeled as luminal cells in the UMAP the keratinocyte-like cells?

Yes – we have corrected the labels in the UMAP in Fig 1B to be consistent with the text.

iii. Figure 1B and 6C need explanation of the annotation for both figures (with feature plots), and the correspondence of clusters (or not) between the Fig 1B and 6C should be discussed.

We have re-labeled the clusters in Fig 6D to align with Fig 1B. Further we provide text to clarify that the goblet cell clusters in the OVX+P4 group are transcriptionally similar to goblet cells clusters in Fig1B.

The setup of the experiment showing persistence and expansion of tdTomato cells after ovx and P4 treatment (Fig S4E) is not clear to me and is in need of a schematic. When were the animals given tamoxifen relative to the ovx, what was the E2 and P4 regimen, and how long after ovx were they characterized?

a. As an aside, this experiment seems highly related to the experiments in figure 6 and perhaps would be best combined with that figure and expanded, as figure 6 claims to characterize "the impact of progesterone on the proliferation and differentiation of the olfm4+ luminal progenitor", but does not perform the relevant lineage tracing to show that this is expansion from the Olfm4+ progenitor rather than conversion of Olfm4- cells into a goblet fate.

We liked the reviewer's suggestion to move the Ovx+P studies in the Olfm4+ TdTomato mice to Figure 6 (see Figure 6C). This also helps to clarify that the same experimental regime used for wild type mice as shown in Fig 6A is used for the Olfm4+ tdTomato line.

3. It is very hard to assess proliferation of Olfm4 positive cells from the data provided in S1C, in part because the Olfm4 staining is so strong on the apical surface and far away from the nuclei.

We have repeated the imaging using Olfm4, KI67 and CDH1. The CDH1 better outlines each cell and we used different colors so that the proliferation of Olfm4+ cells on gestation day 6 is easier to visualize.

4. Figure 1a: presumably the dots in the figure are glands - please describe in the legend or remove.

Figure legend has been revised to clarify the dots are uterine glands.

5. Figure 4a: please update to clarify how many days after tamoxifen injection the diestrus (NP) sample was harvested.

A statement in Figure legend 4A has been added to indicate: The diestrus (NP) samples were collected approximately 1 week after tamoxifen injection.

6. Figure 5A, GD18 WT is spectacular and should be entered into a microscopy competition and/or framed on a wall.

© 2026. Published by The Company of Biologists under the terms of the Creative Commons Attribution License (https://creativecommons.org/licenses/by/4.0/).

THANK YOU! We agree that they are spectacular.

Reviewer 2: SUMMARY OF THE ADVANCE MADE IN THIS PAPER AND ITS POTENTIAL SIGNIFICANCE TO THE FIELD

The manuscript by Madhukaran and coworkers investigates the cell lineage of the mouse cervix. Current dogma states that while the human cervix has two progenitor cells, the columnar and basal cell the mouse cervix has one progenitor cell the basal TP63+ cell. This manuscript shows by immunohistochemistry, single Cell RNA Seq analysis and cell lineage tracing that the mouse has two progenitor cells, The Tp63+ cell and the Krt8+ cell They also demonstrate that the Olfactomedian 4+ , OLFM4+, is a marker for the Krt8+ cell population . They demonstrate that the OLMF4+ cell population is initiated during cervical postnatal development, is increased during pregnancy , induced by progesterone and is a self-renewing population. They also demonstrate that OLMF4 is not required for normal cervical function but serves as a marker for a specific population of cells in the cervix. This report does acknowledge that although it proposes to progenitor cell populations the Tp63+ and The Krt8+ population it does not rule out a Double positive progenitor cell and does acknowledge that lineage tracing with a TP63Cre model is the next step. All in all this is a well designed and executed set of experiment that sets a new paradigm for cervical development in the mouse. It demonstrates that the mouse cervix cell populations align more with the human cervix and presents an in vivo model to investigate the changes in the cervix during pregnancy.

We thank Reviewer 2 for their careful reading of the manuscript and for recognizing the significance of this work in establishing a revised model of mouse cervical epithelial cell lineage. We agree that our finding align the mouse epithelial subtype lineages more closely with the human cervix.

Second decision letter

MS ID#: dev.205710R1

MS TITLE: Olfactomedin 4 marks progenitor cells that give rise to the secretory cell lineage in the mouse cervix

AUTHORS: ShanmugaPriyaa Madhukaran; Yevgenia Fomina; Govindkumar Balagannavar; Elizabeth Payne; Jason Wilson; Lei Wang; Gary Hon; Maria Florian Rodriguez; Mala Mahendroo

Dear Dr Mahendroo,

I have now received the referees report on the above manuscript, and have reached a decision. Their comments are appended below.

The overall evaluation is positive and the Reviewer recommends acceptance. However as you will see they make a few additional points which, if you agree, I feel will further clarify the manuscript and make it easier to follow. The comments are minor and will only involve some modification to the text. We would like to publish a revised manuscript in Development, but would appreciate it if you could make these minor changes before uploading the final version of the paper. As soon as you have uploaded it,I will formally accept the paper and it will be moved through to the production team.

Reviewer 1

© 2026. Published by The Company of Biologists under the terms of the Creative Commons Attribution License (https://creativecommons.org/licenses/by/4.0/).

I very much appreciate the authors' work on the revisions, which I believe have substantially improved the manuscript. It is an excellent contribution to the literature and I support publication.

I have some stylistic suggestions, but they are truly suggestions in case they authors find them valuable and are not required:

1. Running title might read a bit more smoothly with an extra "s" after progenitor i.e. "Olfactomedin4 marks goblet progenitorS"

2. Abstract: I found the abstract a bit dense to read.

a. First sentence: "Female reproductive tract development in mice occurs postnatally." I don't think I understand the importance of this sentence that has warranted its inclusion as the first sentence of the abstract. Also, while this is true, it is somewhat confusing as, of course, much of female reproductive tract development also occurs in utero. I would recommend refining the opening sentence. One option is to use the opening sentence to establish that goblet cells of the cervix are important, as this sets the stage for the impact of the results.

b. The switch from mouse -> human -> mouse is important but a bit confusing, especially without constantly referring to species everywhere:

i. I think it would be clarifying to establish the apparent divergence between mouse and human in a single sentence, e.g. "Current understanding proposes that squamous and secretory cells of the cervicovaginal epithelium arise from a common progenitor in mice, but two distinct progenitors in humans". If this sentence comes early, the rest can switch to mice and stay there until the end.

ii. Add "in mice" to the "here we show" sentence: "Here we identify a population of Krt8+Trp63- epithelial that are retained in the cervicovaginal epithelium during postnatal development and adulthood *in mice*.

c. Last sentence: remove "respectively."

3. I struggled with the way the scRNA seq data (Fig. 1B) was incorporated into the story, even in the revised version although it is much improved. I had 2 struggles.

a. The initial description of the subclassifications of luminal cells as i) goblet cells, ii) a second secretory Muc1 population, and iii) keratinocytes does not make sense to me based on the feature plots. If I were to define 3 groups a priori among the luminal cells just looking at the feature plots, I would define: i) Olfm4+ Muc1+ cells (the "feet"), ii) Olfm4-Muc1- cells ("the legs") and iii) keratinocytes. It doesn't seem to me that the non-goblet secretory (later defined as early goblets) are uniquely defined by being Muc1+.

b. The re-appearance and re-annotation of the data later in the paper is important but as structured ended up being quite confusing for me - we learn that goblet 2 is probably not goblet, and that secretory NP is probably early goblet (although I am surprised that these seem to be Krt8- in the dataset). It was hard to re-orient my brain as these changes were happening.

My recommendation: in Fig 1B (early in the paper), do not yet give the clusters names. Instead, either call them cluster 1, 2, 3, 4 or call them just by their marker annotation e.g. Krt8+Olfm4+Muc1+, Krt8+ Olfm4-Muc1-. Spend a bit more time describing the annotation process. I think dot plots might be easier and more compact than having many feature plots. I think that the markers that would be most informative to show for these initial designations would be: Spdef, Muc5B, Trp63, Krt8, Olfm4, Muc1.

© 2026. Published by The Company of Biologists under the terms of the Creative Commons Attribution License (https://creativecommons.org/licenses/by/4.0/).

Some nested circle/Venn diagram cartoons might be helpful as well to help the reader understand the key points e.g. (i) there are Trp63 and Krt8 cells. (ii) The Krt8+ cells can be sub-classified into Olfm4+ and Olfm4-.

Then, later in the paper you can annotate the clusters as you learn things and put a properly annotated UMAP at the end that the field can take forward.

(A final alternative might be to move these analyses and descriptions entirely to the point where they re-appear in the text rather than the beginning, but then one has to re-engineer the initial narrative about how you arrive at Olfm4).

Second revision

Author response to reviewers' comments

Response to reviewer 1:

We are very appreciative of the helpful suggestions made by the reviewer and agree their suggestions will improve the clarity of the paper. Thus, we have attempted to address the points by revising text and making modifications to Figure 1 and Figure 7. Below we provide point by point comments.

1. Running title might read a bit more smoothly with an extra "s" after progenitor i.e. "Olfactomedin4 marks goblet progenitorS"

Done.

2. Abstract: I found the abstract a bit dense to read.

a. First sentence: "Female reproductive tract development in mice occurs postnatally." I don't think I understand the importance of this sentence that has warranted its inclusion as the first sentence of the abstract. Also, while this is true, it is somewhat confusing as, of course, much of female reproductive tract development also occurs in utero. I would recommend refining the opening sentence. One option is to use the opening sentence to establish that goblet cells of the cervix are important, as this sets the stage for the impact of the results.

b. The switch from mouse -> human -> mouse is important but a bit confusing, especially without constantly referring to species everywhere:

i. I think it would be clarifying to establish the apparent divergence between mouse and human in a single sentence, e.g. "Current understanding proposes that squamous and secretory cells of the cervicovaginal epithelium arise from a common progenitor in mice, but two distinct progenitors in humans". If this sentence comes early, the rest can switch to mice and stay there until the end.

ii. Add "in mice" to the "here we show" sentence: "Here we identify a population of Krt8+Trp63- epithelial that are retained in the cervicovaginal epithelium during postnatal development and adulthood *in mice*.

c. Last sentence: remove "respectively."

We have modified the abstract to address all the above comments and hope the abstract is now easier to read, and the information is more digestible.

3. I struggled with the way the scRNA seq data (Fig. 1B) was incorporated into the story, even in

© 2026. Published by The Company of Biologists under the terms of the Creative Commons Attribution License (https://creativecommons.org/licenses/by/4.0/).

the revised version although it is much improved. I had 2 struggles.

a. The initial description of the subclassifications of luminal cells as i) goblet cells, ii) a second secretory Muc1 population, and iii) keratinocytes does not make sense to me based on the feature plots. If I were to define 3 groups a priori among the luminal cells just looking at the feature plots, I would define: i) Olfm4+ Muc1+ cells (the "feet"), ii) Olfm4-Muc1- cells ("the legs") and iii) keratinocytes. It doesn't seem to me that the non-goblet secretory (later defined as early goblets) are uniquely defined by being Muc1+.

b. The re-appearance and re-annotation of the data later in the paper is important but as structured ended up being quite confusing for me - we learn that goblet 2 is probably not goblet, and that secretory NP is probably early goblet (although I am surprised that these seem to be Krt8- in the dataset). It was hard to re-orient my brain as these changes were happening.

My recommendation: in Fig 1B (early in the paper), do not yet give the clusters names. Instead, either call them cluster 1, 2, 3, 4 or call them just by their marker annotation e.g. Krt8+Olfm4+Muc1+, Krt8+ Olfm4-Muc1-. Spend a bit more time describing the annotation process. I think dot plots might be easier and more compact than having many feature plots. I think that the markers that would be most informative to show for these initial designations would be: Spdef, Muc5B, Trp63, Krt8, Olfm4, Muc1. Some nested circle/Venn diagram cartoons might be helpful as well to help the reader understand the key points e.g. (i) there are Trp63 and Krt8 cells. (ii) The Krt8+ cells can be sub-classified into Olfm4+ and Olfm4-.

Then, later in the paper you can annotate the clusters as you learn things and put a properly annotated UMAP at the end that the field can take forward.
(A final alternative might be to move these analyses and descriptions entirely to the point where they re-appear in the text rather than the beginning, but then one has to re-engineer the initial narrative about how you arrive at Olfm4).

We liked the reviewers suggestion as we too struggled with how best to present this data clearly. We have 1) revised Figure 1 so that the secretory cell clusters are numbered and defined by expression of Tpr63, Krt8 and Olfm4 and we have included a dot plot to better condense the necessary information. In the results section we have provided a description of the annotations and how they are used to define each cluster and 2) revised Figure 7 to include the same UMAP but now with a cell subtype assignment which is supported by the data in the paper. In parallel we have modified text in the Discussion to acknowledge the assignment of early and mature goblet cells.

Third decision letter

MS ID#: dev.205710R2

MS TITLE: Olfactomedin 4 marks progenitor cells that give rise to the secretory cell lineage in the mouse cervix

AUTHORS: ShanmugaPriyaa Madhukaran; Yevgenia Fomina; Govindkumar Balagannavar; Elizabeth Payne; Jason Wilson; Lei Wang; Gary Hon; Maria Florian Rodriguez; Mala Mahendroo

Dear Dr Mahendroo,

I am happy to tell you that your manuscript has been accepted for publication in Development, pending our standard publication integrity checks.

© 2026. Published by The Company of Biologists under the terms of the Creative Commons Attribution License (https://creativecommons.org/licenses/by/4.0/).
